# Bayesian workflow for bias-adjustment model in meta-analysis

**DOI:** 10.1017/rsm.2025.10050

**Published:** 2025-11-13

**Authors:** Juyoung Jung, Ariel M. Aloe

**Affiliations:** Educational Measurement and Statistics, The University of Iowa, United States

**Keywords:** Bayesian meta-analysis, Bayesian workflow, bias adjustment, model validation, risk of bias

## Abstract

Bayesian hierarchical models offer a principled framework for adjusting for study-level bias in meta-analysis, but their complexity and sensitivity to prior specifications necessitate a systematic framework for robust application. This study demonstrates the application of a Bayesian workflow to this challenge, comparing a standard random-effects model to a bias-adjustment model across a real-world dataset and a targeted simulation study. The workflow revealed a high sensitivity of results to the prior on bias probability, showing that while the simpler random-effects model had superior predictive accuracy as measured by the widely applicable information criterion, the bias-adjustment model successfully propagated uncertainty by producing wider, more conservative credible intervals. The simulation confirmed the model’s ability to recover true parameters when priors were well-specified. These results establish the Bayesian workflow as a principled framework for diagnosing model sensitivities and ensuring the transparent application of complex bias-adjustment models in evidence synthesis.

## Highlights

**What is already known?**

- Bayesian models can adjust for bias in meta-analysis, but they are complex, sensitive to prior assumptions, and difficult to apply robustly.
- Applying these models without a clear validation framework can produce misleading results and unwarranted confidence in the findings.

**What is new?**

- We demonstrate a systematic Bayesian workflow to develop and validate a bias-adjustment model designed to handle three common risk-of-bias levels (low, unclear, and high).
- Using both a real-world dataset and a simulation, we demonstrate a workflow that improves transparency and confirms the model’s ability to accurately recover (true) parameters.

**Potential impact for RSM readers**

- The workflow provides a transparent framework for applying complex bias-adjustment models, helping researchers test assumptions and improve the credibility of their findings.
- This approach helps produce more robust and defensible conclusions when bias is a concern, encouraging wider adoption of these advanced methods in evidence synthesis.

## 1 Introduction

Meta-analysis synthesizes quantitative findings from multiple studies to inform decision-making across diverse fields, including education, clinical practice, and health policy. By increasing statistical power and precision, it provides evidence summaries that extend beyond the limitations of individual trials.^1^ However, the validity and reliability of meta-analytic conclusions depend critically on addressing two fundamental challenges that threaten evidence synthesis: between-study heterogeneity and systematic bias. While heterogeneity is routinely handled through random-effects models, systematic bias remains a more complex and methodologically demanding problem. When biased studies systematically over- or under-estimate true treatment effects, meta-analytic conclusions can be distorted, producing misleading evidence that may misinform decisions.^2^^,^ ^3^

Bias arises from multiple sources, including methodological flaws, such as inadequate randomization, lack of blinding, selective outcome reporting, and attrition, as well as selective dissemination of results and the inclusion of lower-quality studies.^4^^,^ ^5^ To support structured evaluation, tools, such as the Cochrane Risk of Bias 2 (RoB2) for randomized trials^6^ and ROBINS-I for observational studies,^7^ classify studies into risk of bias categories (e.g., low, unclear/some concerns, and high) across multiple domains. However, identifying and classifying the risk of bias is only a preliminary step; the critical challenge is moving from this qualitative assessment to quantitative bias adjustment. This involves incorporating bias evaluations directly into the meta-analytic model to adjust effect estimates and properly account for uncertainty about the magnitude and prevalence of bias.^8^^,^ ^9^

Bayesian hierarchical modeling provides a principled framework for such adjustments. These approaches explicitly represent the bias mechanism through mixture distributions that attempt to separate true underlying treatment effects ( 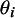 ) from systematic distortions introduced by bias ( 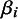 ). Observed effects in potentially biased studies ( 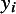 ) can thus be expressed as 
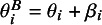
 , where 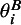 denotes the biased latent effect.^10^^–^ ^12^ Bayesian methods are particularly advantageous because they allow for the incorporation of external information via priors, enable the simultaneous estimation of both bias-adjusted treatment effects and their associated uncertainty, and facilitate principled down-weighting of studies according to bias risk rather than relying on arbitrary exclusion criteria.

Despite these advantages, implementing bias-adjusted meta-analysis models presents substantial methodological challenges. A central difficulty lies in balancing the introduction of bias-related parameters with the preservation of realistic uncertainty. Model specification requires careful attention to bias mechanisms, prior distributions, and identifiability constraints, while limited data may cause posterior distributions to be heavily driven by prior assumptions.^13^ These difficulties can lead to misleading inferences.^14^^–^ ^18^ For example, poorly chosen priors may yield paradoxical results, such as overly narrow credible intervals despite the inclusion of additional parameters, thereby creating unwarranted confidence in biased evidence. Such risks underscore the importance of robust modeling strategies that can diagnose, evaluate, and prevent misspecification.^19^^,^ ^20^

The Bayesian workflow can offer a structured, transparent framework for systematic model development that addresses these challenges through iterative specification, diagnostic evaluation, and refinement.^21^ Rather than treating model fitting as a one-step procedure, the workflow emphasizes the full process of model building and interpretation. Prior predictive checks help determine whether assumed distributions for bias and heterogeneity yield plausible data patterns before fitting the model. Sensitivity analysis can highlight how strongly conclusions depend on assumptions about the likelihood of bias. Posterior predictive checks then evaluate whether the fitted model adequately reproduces key features of the observed data, thereby diagnosing potential misspecifications. Model comparison further supports the selection of specifications that best balance empirical fit with theoretical plausibility.^22^^–^ ^24^ Taken together, these components enhance transparency in modeling decisions, strengthen the robustness of bias adjustment, and improve the credibility of resulting inferences.

This study bridges the gap between advanced methodology and its practical application using both a real-world meta-analysis and a (targeted) simulation data. While bias-adjustment models are available, their use in practice is often incomplete, focusing on final estimates without the model validation needed to ensure reliable conclusions. Many demonstrations of the Bayesian workflow, conversely, use simpler models, leaving a gap in how to apply these principles to complex meta-analytic problems. Specifically, we show how an iterative workflow guides critical decisions in prior specification and model evaluation, safeguarding against misspecification and enhancing the credibility of inferences. We apply this systematic process to an extended existing bias-adjustment model by Verde^11^ that incorporates three levels of risk-of-bias classification (“low,” “unclear,” and “high”), aligning it with widely used tools, such as RoB2 and ROBINS-I. The results from both the applied example and the simulation illustrate how embedding bias adjustment within this systematic process produces conclusions that are more nuanced, resistant to distortion from flawed studies.

## 2 Bayesian bias-adjustment meta-analysis model

We introduce a Bayesian hierarchical model for meta-analysis that incorporates study-level risk of bias assessments, extending the framework of Verde.^11^ Unlike approaches that rely on study design (e.g., randomized controlled trial vs. observational study) as a proxy for bias, our model directly integrates risk-of-bias classifications (low, unclear, and high), acknowledging that bias can occur across all study types.^25^^–^ ^27^

### 2.1 Model specification

Suppose a meta-analysis includes *N* studies. For study *i*, for 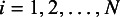 , let 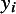 denote the observed (reported) effect size (e.g., standardized mean difference and log odds ratio) with known (or well-estimated) standard error 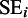 . We model (2.1)

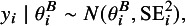

where 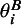 is the potentially biased effect size for study *i*.

To account for bias, the core of this model is the decomposition of 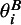 into an unbiased treatment effect ( 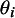 ) and an additive bias term ( 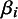 ), modeled as a mixture (2.2)

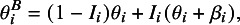

where 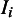 is a latent indicator of whether study *i* is biased. If 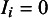 (unbiased), the effect is simply the true effect 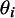 ; if 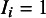 (biased), it becomes the true effect plus a bias term, 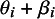 .

### 2.2 Risk of bias level integration

The model framework extends to accommodate studies with an “unclear” risk-of-bias rating, which are common in systematic reviews and introduce additional uncertainty. For these studies, the bias indicator, 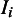 , is not treated as a fixed value but rather as a random variable to formally model this uncertainty as defined (2.3)

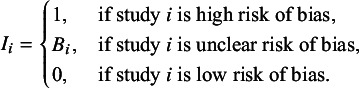



Specifically, for a study with an unclear risk-of-bias rating, its bias status is determined by a Bernoulli process as (2.4)

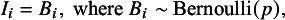

where 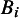 is a latent variable representing the study’s true (but unknown) bias status, and *p* is the probability that a study rated as “unclear” is, in fact, biased. This probabilistic approach allows the model to determine the bias status of unclear studies based on both the data and the prior information supplied for *p*.^28^ A common choice is to set 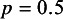 , which reflects a state of maximum uncertainty about the bias status of unclear studies. This default acknowledges that an “unclear” rating often implies insufficient information to make a definitive judgment, making equal probabilities of the study being biased or unbiased a starting point.

### 2.3 Hierarchical model for effects and bias

The true effect sizes 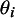 and bias term 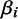 are modeled hierarchically as (2.5)

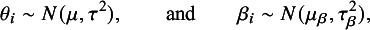

where 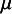 is the overall mean effect, 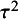 is the between-study heterogeneity variance, 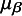 is the biased mean across only for biased studies, and 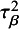 is the between-study variance in bias. Identifiability is ensured by assuming a common bias mean ( 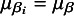 ) and typically a positive bias direction ( 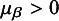 ), based on contextual evidence.

A parameter in the model is 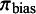 (i.e., 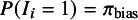 ), the overall probability that a study is biased. A significant challenge in bias-adjustment models is the weak identifiability of this parameter, as the available data often provide limited information to distinguish between variance arising from true between-study heterogeneity ( 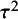 ) and variance attributable to bias ( 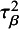 ). Consequently, the posterior distribution of 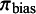 can be highly sensitive to its prior specification. To address this, we move beyond default or uninformative priors and instead assign an informative Beta distribution, which is mathematically suited for modeling probabilities on a 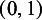 scale as follows: (2.6)

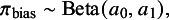

where the hyperparameters 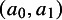 of this distribution are not chosen arbitrarily but are calibrated using empirical information derived directly from our risk-of-bias assessments. This is achieved by anchoring the prior distribution at two quantiles, which allows us to transparently encode our beliefs about the prevalence of bias.

The first anchor establishes a plausible upper bound for bias prevalence. We set the 90th percentile of the prior distribution equal to the observed proportion of studies rated as having a high risk of bias 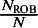 . The rationale for this is that the true proportion of biased studies in the meta-analysis is unlikely to be substantially greater than the proportion of studies already identified with clear methodological flaws. This constraint prevents the model from exploring unrealistically high values for 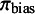 . The second anchor sets the median, or the 50th percentile, of the prior distribution. This anchor incorporates a crucial skepticism parameter, *K*, which allows us to express our degree of confidence in the risk-of-bias ratings themselves as follows: (2.7)

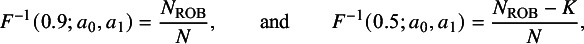

where *K* explicitly acknowledges that a “high risk of bias” rating does not perfectly and invariably translate to a biased effect size. In fact, empirical evidence suggests that the link between specific risk-of-bias domains and the magnitude of effect sizes can be inconsistent. The value of *K* adjusts the prior accordingly to reflect this uncertainty. For instance, a small *K* (e.g., 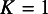 ) reflects strong confidence in the assessments, positioning the median very close to the upper bound and implying a strong prior belief that nearly all studies rated as “high risk” are truly biased. Conversely, a larger *K* reflects greater skepticism by shifting the median lower and creating a more diffuse prior, which gives more weight to the possibility that some studies flagged as “high risk” may have nonetheless produced unbiased estimates.

### 2.4 Variance partitioning and mixture distribution

Following Verde,^11^ we introduce a rigorousness weight *q* to partition total variance into heterogeneity and bias components as defined (2.8)

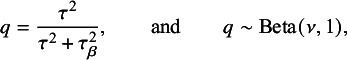

where *q* represents the proportion of total variance that is attributable to between-study heterogeneity ( 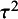 ) rather than bias ( 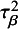 ). It is important to note that the model also specifies a separate prior for the heterogeneity, 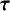 . The bias variance, 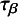 , is not assigned its own independent prior; instead, it is a derived parameter determined jointly by the priors on *q* and 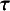 through the relationship. In the prior for the *q*, a smaller value for the shape parameter 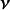 (e.g., 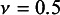 ) more strongly discounts the contribution of studies deemed less trustworthy by yielding a lower average weight.

Integrating over bias, the biased effect distribution is expressed as a mixture (2.9)

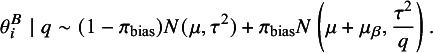



This formulation captures both the variability among unbiased studies and the additional variation due to bias. The resulting distribution features heavier tails, resembling a slash distribution,^29^ which enhances robustness against outliers. By incorporating a slash distribution—characterized by heavier tails than a normal distribution and symmetry around its mean, with location 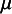 , scale 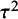 , and shape 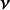 for 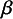 —the model accounts for uncertainty in the direction of bias.

## 3 Overview of Bayesian workflow

The Bayesian workflow provides a structured framework for statistical modeling that emphasizes an iterative process of model specification, fitting, checking, and refinement.^21^ The workflow begins with model specification, where a full probability model is defined. This involves selecting a likelihood function, 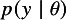 , which describes the data-generating process for the observed data 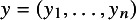 given the parameters 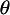 , and choosing a prior distribution, 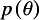 , which quantifies pre-existing knowledge or assumptions about these parameters. In a bias-adjustment meta-analysis context, 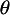 would encompass all relevant parameters, potentially including study-specific effects 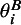 , an overall effect 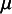 , heterogeneity 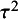 , and bias-related parameters to 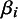 .

Before fitting the model to the actual data, prior predictive checks are performed. These involve simulating datasets 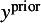 from the joint prior predictive distribution 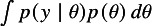 to understand the a priori implications of the model and priors. Comparing these simulations against domain expertise helps identify unrealistic assumptions early on. Following the specification, the model is fitted to the observed data to compute the posterior distribution, 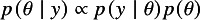 , typically using computational techniques (e.g., Markov chain Monte Carlo [MCMC]). Ensuring the reliability of this computation is critical, requiring computational diagnostics, such as checking MCMC chain convergence (e.g., verifying that the potential scale reduction factor 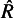 is close to 1) and assessing effective sample sizes.

Once posterior samples are obtained, posterior predictive checks are essential for evaluating model adequacy. Replicated datasets 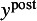 are simulated from the posterior predictive distribution 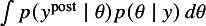 , and their properties are compared to the observed data *y*. Graphical comparisons (e.g., density overlays) and comparisons of test statistics (e.g., means and standard deviation) help diagnose systematic misfits between the model and the data. In parallel, sensitivity analysis investigates the robustness of conclusions by varying model assumptions, particularly the prior distributions for monitored parameters, and observing the impact on posterior inferences.

Model comparison techniques are employed when evaluating or comparing different candidate models. Information criteria provide a valuable tool by estimating pointwise out-of-sample prediction accuracy, effectively balancing model fit against complexity. Widely applicable information criterion (WAIC), a measure of predictive accuracy that balances model fit and complexity in Bayesian models, is calculated from posterior simulations 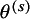 ( 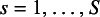 ) using the log pointwise predictive density (LPPD), which quantifies model fit as the sum of the log predictive densities for each data point averaged over posterior simulations, and an effective parameter count penalty (pWAIC), which adjusts for model complexity by estimating the effective number of parameters based on the variance of the log predictive densities, via (3.1)

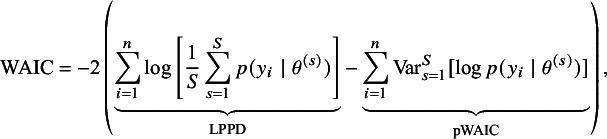

where lower WAIC values indicate stronger predictive performance. Conversely, higher WAIC values suggest a poorer trade-off between model fit and complexity, indicating weaker predictive accuracy.^30^

## 4 Data source

Data for this study were obtained from an openly accessible repository at https://osf.io/fby7w/, from the meta-analysis titled *The Effects of Co-Teaching and Related Collaborative Models of Instruction on Student Achievement*.^31^ The original meta-analysis synthesized evidence on co-teaching and related collaborative instructional models, evaluating their impact on student achievement. Co-teaching, broadly defined, involves two or more educators jointly delivering instruction to a group of students, often as part of inclusion practices for students with disabilities, though applications extend to diverse educational settings. The interventions compared in the primary studies typically contrasted co-teaching or collaborative models (e.g., team teaching, station teaching, and parallel teaching) with business-as-usual instruction or other less collaborative instructional formats. The primary outcome across studies was student academic achievement, measured through standardized test scores, curriculum-based assessments, or teacher-constructed achievement tests. The populations represented in the meta-analysis were predominantly K–12 students, across both general education and special education contexts, reflecting the wide application of co-teaching practices in inclusive classrooms.

The full dataset includes 280 effect sizes from 76 unique studies. To avoid dependence between multiple effect sizes from the same study, we extracted a subset of the dataset by selecting one effect size per unique study, yielding 76 effect sizes for analysis. We used unadjusted Hedges’ *g* effect sizes with standard errors ( 
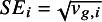
 ) as provided in the original dataset. Risk of bias assessments in the original meta-analysis, conducted using the RoB2 and ROBINS-I tools, categorized studies into five levels (“low,” “moderate,” “some concerns,” “serious,” and “high”). For our modeling, we recoded these into three levels—“low,” “unclear,” and “high”—to align with our bias-adjustment framework. “Moderate” and “some concerns” were merged into “unclear,” while “serious” and “high” were combined into “high.” In our data subset, the proportions of these risk-of-bias levels were 2.63% “low,” 43.40% “unclear,” and 53.90% “high.” These recoded ratings were then converted into a binary indicator, 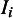 . After recoding, 23.68% of the effect sizes were assigned 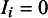 (low or unclear), and 76.32% were assigned 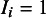 (high or unclear). For studies with an “unclear” rating, the indicator 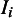 was randomly assigned using a Bernoulli distribution ( 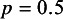 ) to reflect uncertainty about their true bias status.

## 5 Statistical analysis

Our statistical analysis followed a Bayesian workflow to specify, fit, and evaluate two meta-analysis models: a standard random-effects model and a bias-adjustment model. The workflow consisted of four stages: prior predictive checks to assess the plausibility of prior assumptions, model fitting using MCMC, posterior predictive checks to evaluate how well models captured observed data features, and model comparison using predictive accuracy criteria.

### 5.1 Model specifications

Two Bayesian meta-analysis models are specified. In the random-effects model, the likelihood is given by 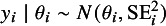 , where 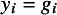 represents the observed effect size for study 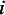 . The study-specific true effects 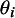 are assumed to be drawn from a common normal distribution: 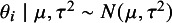 . In the bias-adjustment model, the likelihood is given by 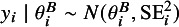 . The study-specific precision 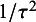 depends on the bias status 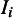 and a weight parameter 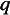 , which is applied only to biased studies. If 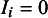 , then the precision is 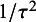 ; otherwise, if 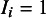 , it becomes 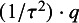 . The overall probability of bias, 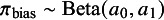 , is subject to sensitivity analysis.

### 5.2 Prior predictive checks

Each simulation ( 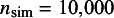 ) replicated the study count ( 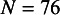 ) and incorporated the observed study-specific standard errors ( 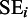 ) to reflect realistic measurement precision. For the random-effects model, we specified priors of 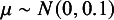 and 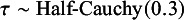 . These weakly informative priors encode the expectation, supported by prior educational research, that the overall effect is likely small and that heterogeneity is moderate, while still allowing for substantial between-study variation.^32^^,^ ^33^ For the bias-adjustment model, we used 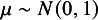 for the mean effect of unbiased studies and assigned the bias magnitude a broad prior of 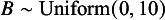 . This specification acknowledges the possibility of both small and large upward distortions without imposing restrictive constraints.

Under this setup, the expected prior predictive mean can be derived as 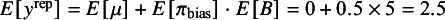 , where 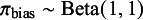 reflects maximum uncertainty about the prevalence of bias, and 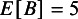 follows from the midpoint of the uniform prior. This calculation highlights the implications of the joint prior specification for expected effect sizes. The shared heterogeneity standard deviation was assigned 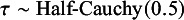 , reflecting a weakly informative belief that heterogeneity in educational interventions is likely moderate, while permitting heavier-tailed uncertainty. For each simulation, a bias indicator 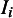 was drawn from 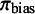 . If 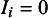 , the effect was drawn from 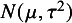 . If 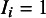 , the model introduced additional variability by first sampling a rigorousness weight 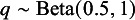 , then drawing the biased effect from a slash distribution centered at 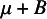 with variance adjusted by 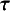 and 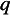 . The slash distribution was chosen for its heavier tails compared to a normal distribution, improving robustness to extreme distortions often found in biased studies. Final observed values 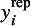 were then generated by adding sampling error via 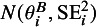 .

### 5.3 Model fitting

Both models were estimated using MCMC in JAGS^34^ via R^35^ with four parallel chains of 200,000 iterations, discarding the first 40,000 as burn-in and retaining every 10th draw, resulting in 64,000 posterior samples per parameter. Convergence was confirmed by 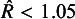 for all monitored parameters. For the bias-adjustment model, we conducted a sensitivity analysis on the prior for the bias prevalence parameter, 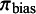 , to reflect differing assumptions about the proportion of biased studies. Priors were calibrated so that the prior median probability of bias was set at 0.55, 0.60, 0.65, or 0.70, with the 90th percentile anchored at the observed proportion of high-risk studies.

These targets yielded the following Beta parameterizations corresponding to tuning levels 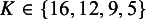 : ( 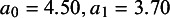 ); ( 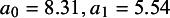 ); ( 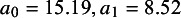 ); and ( 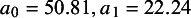 ). Smaller *K* values imply a stronger belief that most high-risk studies are truly biased, whereas larger *K* reflect greater skepticism about bias prevalence. The prior for the bias magnitude was specified as 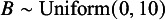 , providing a broad but reasonable range for potential distortions. Priors for the overall mean effect and heterogeneity were consistent with those used in the prior predictive checks, ensuring coherence between prior exploration and model fitting.

### 5.4 Model evaluation

For each fitted model, replicated datasets 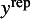 were generated by drawing from the posterior predictive distribution, 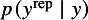 . Each 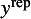 represents a dataset of effect sizes that could plausibly have been observed if the fitted model were the true data-generating process. These replicated datasets were then compared to the observed dataset 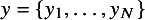 . Two types of comparisons were conducted: 1) density overlay plots were generated to visually compare the estimated density of the observed data 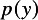 with the densities of numerous replicated datasets 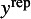 and 2) specific summary statistics were chosen as discrepancy measures to check if the model captures particular features of the data. Following the analysis, the sample mean ( 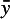 ) and the sample standard deviation ( 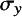 ) of the effect sizes were used. The observed values of these statistics ( 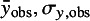 ) were compared to the distributions of the same statistics calculated from the replicated datasets ( 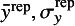 ).

### 5.5 Model comparison

The WAIC was calculated from the posterior results of each fitted model to aid in model evaluation and comparison. For each model, WAIC was computed as 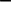 2 times the LPPD plus twice the effective number of parameters, using an 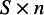 log-likelihood matrix (*S* posterior draws and *n* data points). Differences in WAIC values between models were estimated, and standard errors were calculated to assess uncertainty in the comparisons. The model with the lowest WAIC was identified for subsequent analyses, including parameter estimation and predictive inference.

## 6 Results

**Figure 1:**
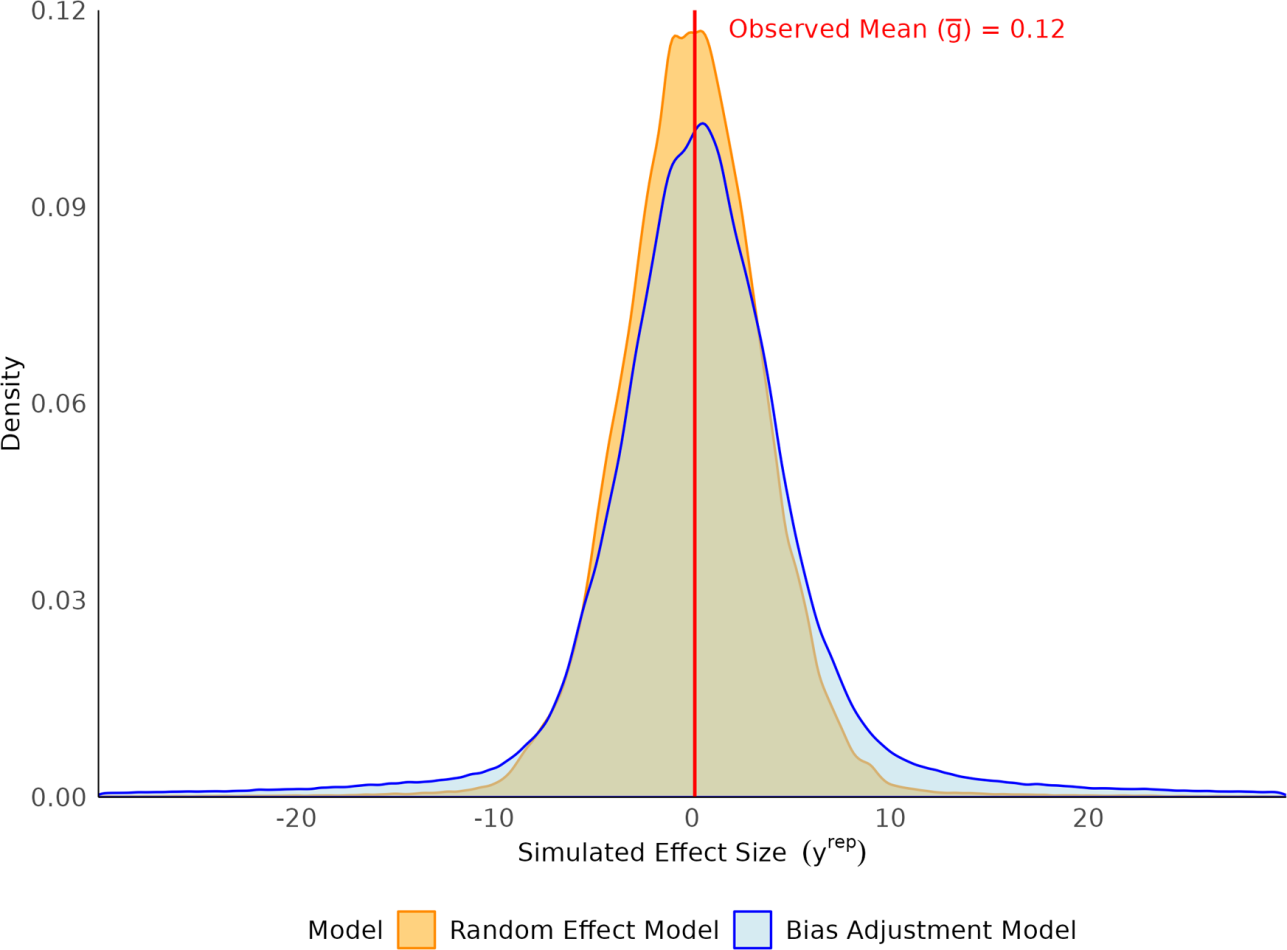
Prior predictive distributions of simulated effect sizes for random-effect and bias-adjustment models.

**Figure 2:**
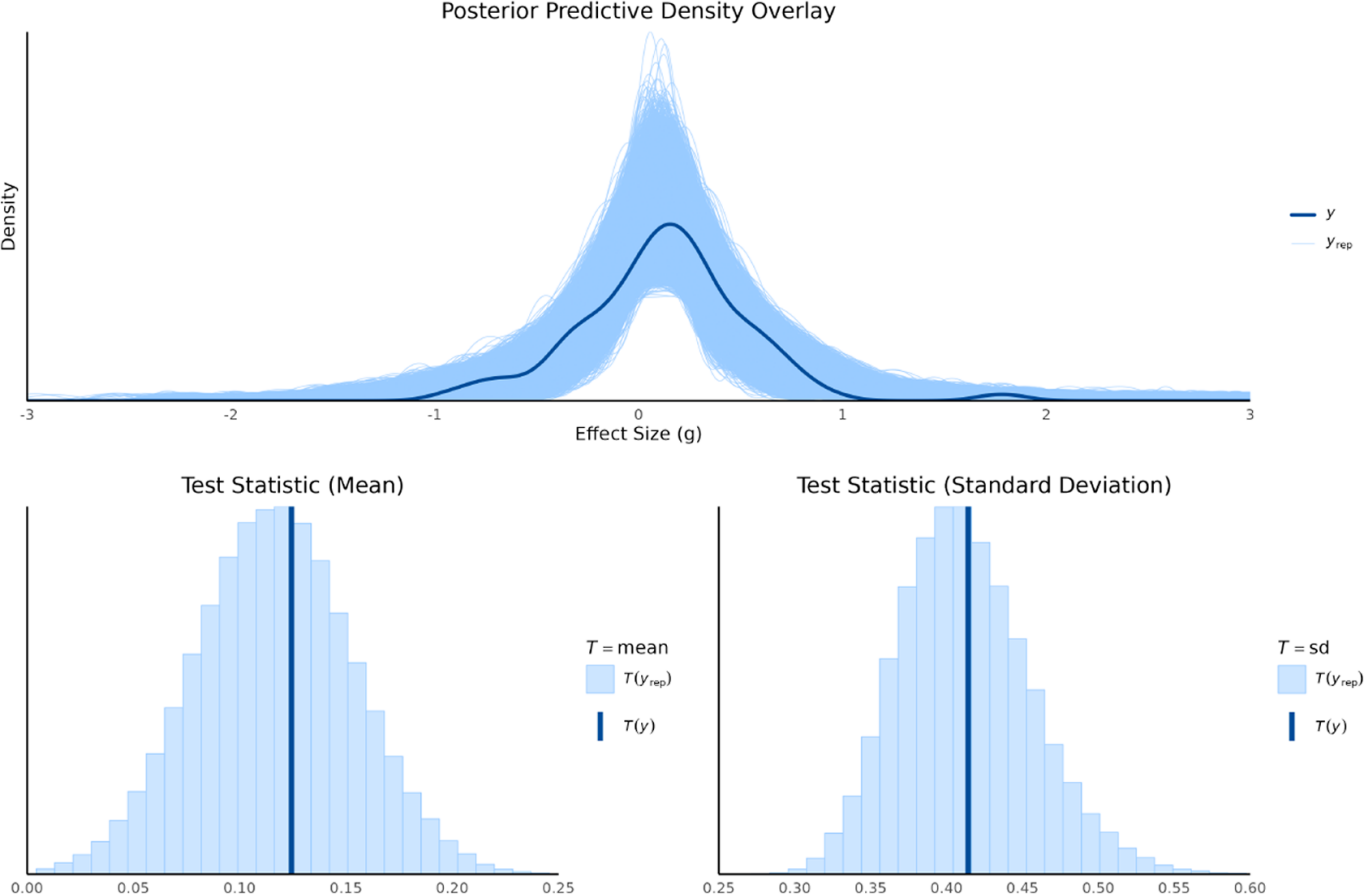
Posterior predictive density overlay and test statistics for random-effect model.

**Figure 3:**
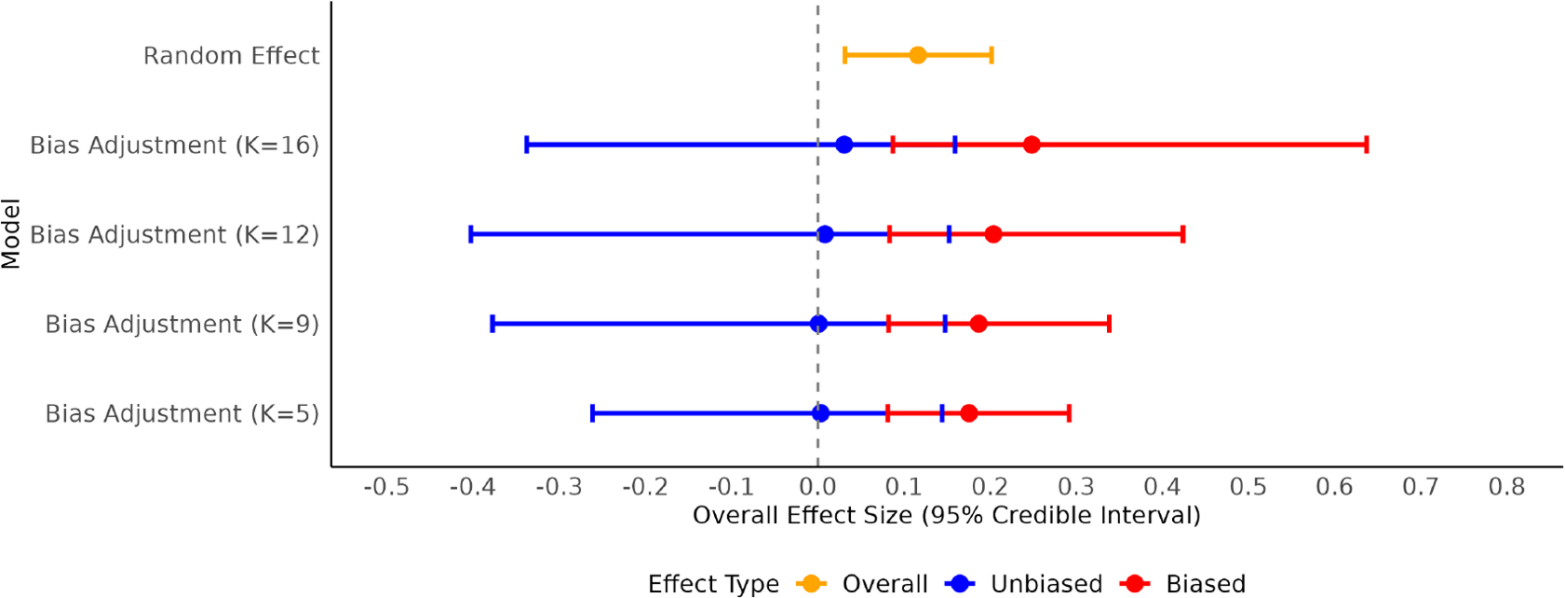
Overall effect size forest plot for random-effect and bias-adjustment models.

**Figure 4:**
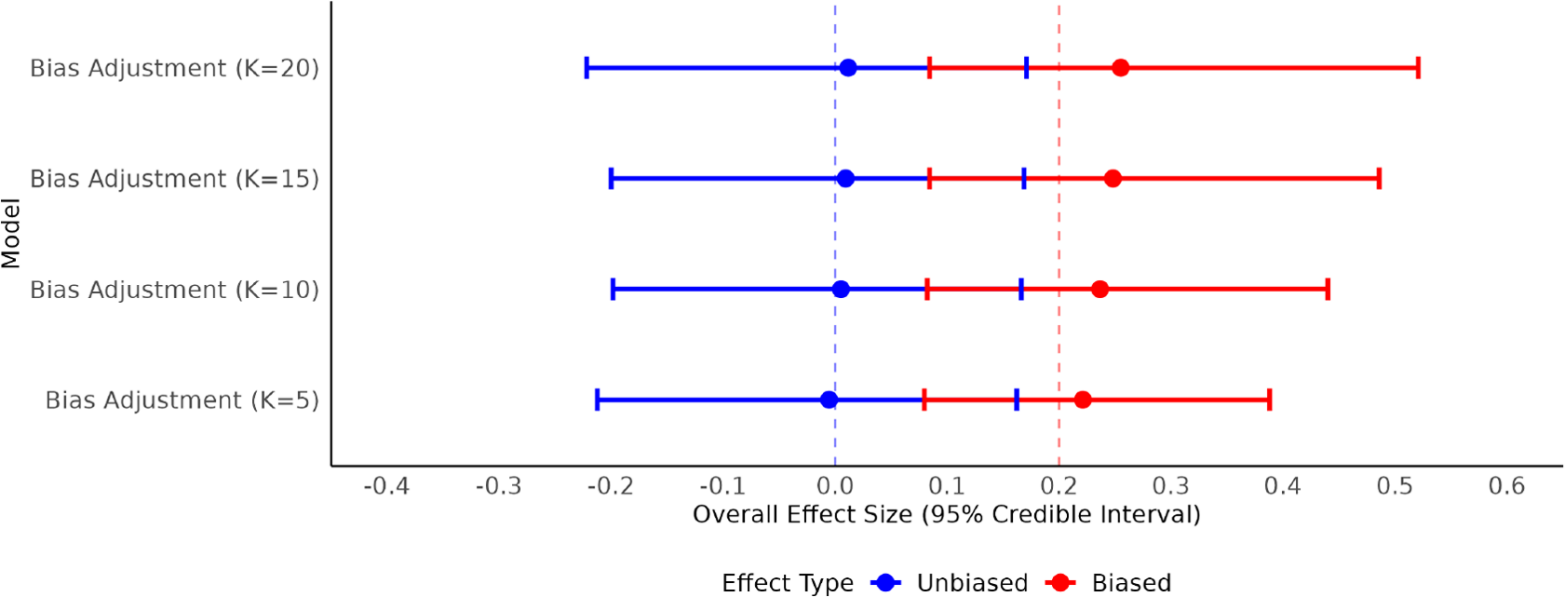
Overall effect size forest plot for bias-adjustment models with simulation data.

**Figure A1:**
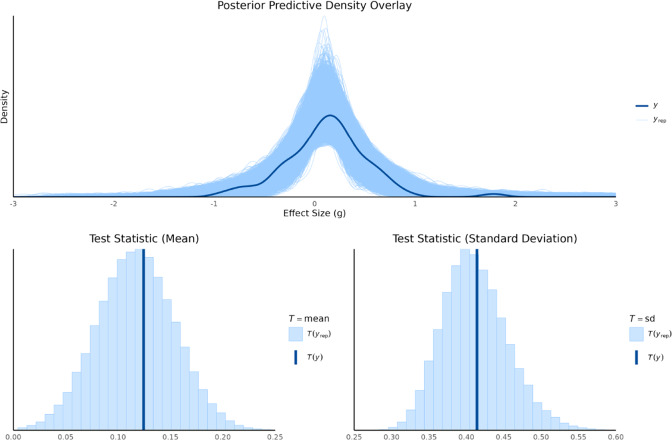
Posterior predictive density overlay and test statistics for bias-adjustment model ( 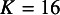 ).

**Figure A4:**
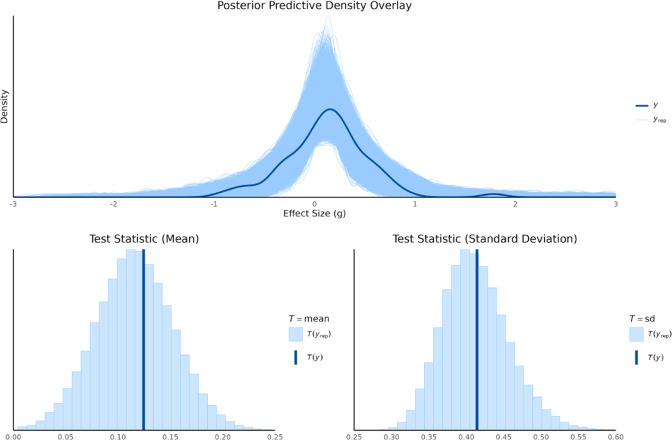
Posterior predictive density overlay and test statistics for bias-adjustment model ( 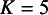 ).

**Figure A5:**
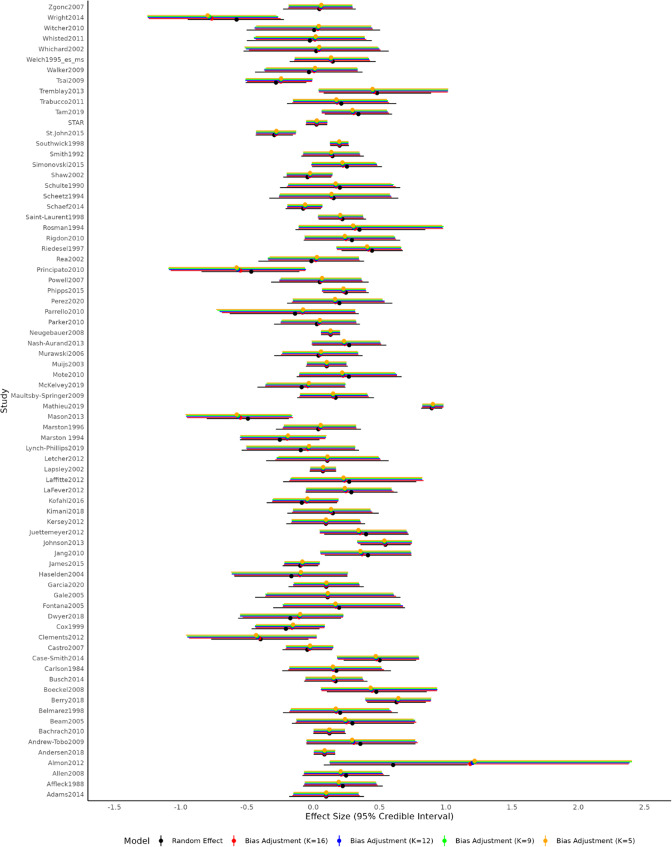
Study-specific effect size forest plot for random-effect and bias-adjustment models.

The primary results of the comparative model fitting are illustrated in the main text (Figures 1–4), which show the prior predictive distributions used to evaluate assumptions, the posterior predictive fit of the random-effects model, and forest plots summarizing the overall effect sizes for both the empirical and simulated data. The Appendix provides additional figures showing the posterior predictive checks for each of the bias-adjustment model sensitivity analyses (Figures A1–A4). A detailed forest plot is also included in the Appendix, which displays the individual effect size estimates and 95% credible intervals for every study across all fitted models (Figure A5). The R code^35^ used for the statistical analyses and generation of all figures is available in the Supplementary Material, ensuring full reproducibility of the findings.

### 6.1 Results of prior predictive checks

We used prior predictive simulation to evaluate whether the specified priors, together with the likelihood, imply plausible meta-analytic effect sizes before seeing the data. For the random-effects model with 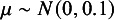 and 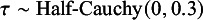 , the implied distribution of replicated effect sizes 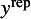 was symmetric and comparatively narrow, consistent with modest heterogeneity as shown in Figure 1. For the bias-adjustment model, which introduces a mixture structure with bias magnitude *B*, variance-partition weight *q*, and bias prevalence 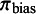 , together with a slash distribution for the biased component, the prior predictive distribution was broader with heavier tails. Intuitively, 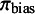 controls the mixing weight on the biased component, *q* inflates the variance of that component via 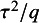 , and *B* shifts its location; diffuse choices for any of these push probability mass into the extremes.

The empirical summaries of the observed effects, the observed mean effect size ( 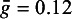 ) with range 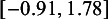 and standard deviation (0.41) lay well within both models’ prior predictive envelopes, indicating that the baseline priors on 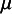 and 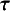 are compatible with the scale of the literature. However, under diffuse bias priors, specifically 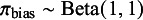 and a wide bias-magnitude prior 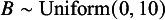 , the implied mean shift for biased studies is 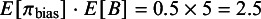 , which is implausibly large on the Hedges’ *g* scale for education interventions. This combination, together with the heavy-tailed slash distribution, generated replicated datasets whose dispersion exceeded that of the observed effects, reflected in a broad, low-peaked prior predictive density.

Guided by these diagnostics, and by the observed distribution of risk-of-bias ratings in the dataset, we replaced the uninformative 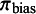 prior with an informative 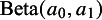 calibrated to the proportion of studies rated high risk (anchoring the 90th percentile at 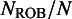 and setting the prior median at 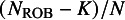 ; see Equation (2.7)), and constrained *B* to a more conservative range in the prior-checking stage. These adjustments retain the model’s capacity to represent substantial bias when warranted, while aligning the implied 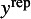 distribution with historically plausible effect sizes and the study-level risk-of-bias information. The resulting prior predictive distributions remained centered near zero, covered the empirical summaries, and exhibited tail behavior commensurate with the application domain rather than dominated by extreme, a priori unlikely shifts.

### 6.2 Results of model fitting

The effect size in this analysis is Hedges’ *g*, representing the impact of co-teaching on student academic achievement; positive values indicate a benefit over traditional single-teacher instruction. Both the random-effects model and the bias-adjustment variants achieved satisfactory convergence ( 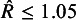 for all parameters). Table 1 summarizes the posterior estimates for parameters, including mean effects for unbiased and biased studies ( 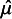 and 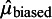 ), heterogeneity ( 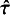 ), bias magnitude ( 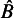 ), and the posterior probability of bias ( 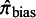 ), revealing systematic patterns in how bias-adjustment affects parameter estimates and uncertainty quantification. The random-effects model yielded an overall mean effect of 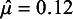 (95% CrI: [0.03, 0.20]; SD = 0.04), suggesting a small positive effect of co-teaching. The heterogeneity estimate was 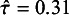 (95% CrI: [0.25, 0.39]; SD = 0.04), indicating moderate between-study variability.

**Table 1:** Posterior summaries for random-effect and bias-adjustment models

Parameter	Mean	Standard deviation	95% Credible interval	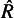
*Random-effect model*				
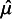	0.12	0.04	[0.03, 0.20]	1.00
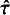	0.31	0.04	[0.25, 0.39]	1.00
*Bias-adjustment model* ( 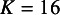 )				
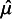	0.03	0.11	[ 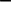 0.34, 0.16]	1.01
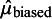	0.25	0.17	[0.09, 0.64]	1.02
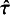	0.16	0.06	[0.06, 0.29]	1.00
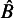	0.22	0.18	[0.01, 0.62]	1.00
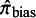	0.49	0.21	[0.13, 0.88]	1.00
*Bias-adjustment model* ( 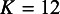 )				
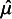	0.01	0.14	[ 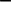 0.40, 0.15]	1.02
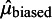	0.20	0.09	[0.08, 0.42]	1.00
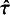	0.13	0.05	[0.06, 0.24]	1.00
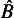	0.20	0.15	[0.01, 0.56]	1.00
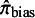	0.59	0.16	[0.29, 0.87]	1.00
*Bias-adjustment model* ( 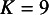 )				
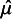	0.00	0.13	[ 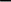 0.38, 0.15]	1.01
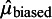	0.19	0.07	[0.08, 0.34]	1.00
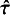	0.12	0.04	[0.06, 0.20]	1.01
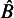	0.19	0.14	[0.01, 0.54]	1.00
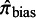	0.65	0.11	[0.43, 0.84]	1.00
*Bias-adjustment model* ( 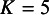 )				
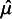	0.00	0.10	[ 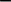 0.26, 0.14]	1.01
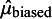	0.18	0.05	[0.08, 0.29]	1.00
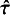	0.11	0.03	[0.06, 0.18]	1.00
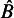	0.17	0.12	[0.01, 0.45]	1.00
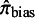	0.70	0.06	[0.59, 0.80]	1.00

*Note*: 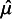 = unbiased mean effect; 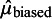 = biased mean effect; 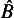 = (mean) bias magnitude; 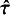 = heterogeneity standard deviation; 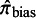 = (posterior) probability of bias; 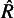 = potential scale reduction factor.

For the bias-adjustment models with prior specifications indexed by 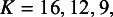 and 5, the results revealed systematic differences in parameter estimates. Under 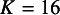 , the unbiased mean effect was 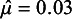 (95% CrI: [ 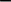 0.34, 0.16]; SD = 0.11), while the biased mean was 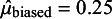 (95% CrI: [0.09, 0.64]; SD = 0.17), producing an estimated bias magnitude of 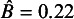 (95% CrI: [0.01, 0.62]; SD = 0.18). The posterior probability of bias was 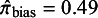 (95% CrI: [0.13, 0.88]; SD = 0.21), with heterogeneity reduced to 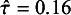 (95% CrI: [0.06, 0.29]; SD = 0.06). When the prior became more informative at 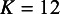 , the unbiased mean decreased slightly to 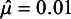 (95% CrI: [ 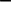 0.40, 0.15]; SD = 0.14), the biased mean remained positive at 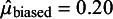 (95% CrI: [0.08, 0.42]; SD = 0.09), and the bias magnitude narrowed to 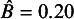 (95% CrI: [0.01, 0.56]; SD = 0.15). At the same time, the probability of bias rose to 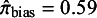 (95% CrI: [0.29, 0.87]; SD = 0.16), with heterogeneity further reduced to 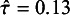 (95% CrI: [0.06, 0.24]; SD = 0.05).

This pattern continued as *K* decreased. At 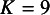 , the unbiased mean was essentially null ( 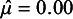 ; 95% CrI: [ 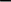 0.38, 0.15]; SD = 0.13), while the biased mean remained positive at 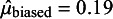 (95% CrI: [0.08, 0.34]; SD = 0.07). The estimated bias magnitude was 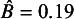 (95% CrI: [0.01, 0.54]; SD = 0.14), with the probability of bias increasing to 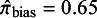 (95% CrI: [0.43, 0.84]; SD = 0.11) and heterogeneity declining to 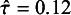 (95% CrI: [0.06, 0.20]; SD = 0.04). Finally, under the most informative prior at 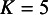 , the unbiased mean remained near zero ( 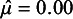 ; 95% CrI: [ 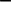 0.26, 0.14]; SD = 0.10), while the biased mean was 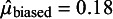 (95% CrI: [0.08, 0.29]; SD = 0.05). The bias magnitude estimate narrowed to 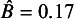 (95% CrI: [0.01, 0.45]; SD = 0.12), the probability of bias rose to 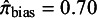 (95% CrI: [0.59, 0.80]; SD = 0.06), and residual heterogeneity decreased further to 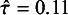 (95% CrI: [0.06, 0.18]; SD = 0.03).

The sensitivity analysis revealed that as the prior for the probability of bias became more informative (from 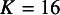 to 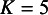 ), the model systematically re-attributed variance from random heterogeneity to systematic bias. This re-partitioning had two main consequences. First, the unbiased effect estimate ( 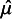 ) was adjusted progressively toward zero, while the biased effect estimate remained positive. Second, this process correctly propagated uncertainty, resulting in wider, more conservative credible intervals for the unbiased effect compared to the standard random-effects model, as the heterogeneity estimate ( 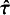 ) decreased from 0.16 to 0.11. A finding was the model’s high sensitivity to the bias probability prior ( 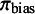 ). Its posterior estimate was heavily influenced by the prior choice, with the 95% credible interval shrinking dramatically as the prior became more informative (from a width of 0.75 at 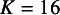 to 0.21 at 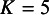 ). This demonstrates that stronger priors can dominate the data, underscoring the critical importance of carefully justified prior specification in bias-adjustment models.

### 6.3 Results of model evaluation

Posterior predictive checks were conducted to evaluate how well the fitted models captured the features of the observed data. This was done by comparing the distribution of the observed data (*y*) to distributions of replicated data ( 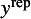 ) drawn from each model’s posterior predictive distribution. We used both graphical density overlays and comparisons of summary statistics (mean and standard deviation). For the random-effects model, shown in Figure 2, the replicated data closely mirrored the observed data’s density, indicating a good overall fit. The observed mean ( 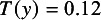 ) and standard deviation ( 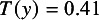 ) fell squarely within the center of their respective replicated distributions, confirming that the model effectively captures both the central tendency and the variability of the data.

The bias-adjustment models ( 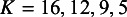 ; Figures A1–A4) similarly reproduced the observed data distributions. Across all priors, the observed mean ( 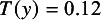 ) consistently fell near the center of the replicated mean distributions, showing that adjustment for bias did not compromise the models’ ability to capture central tendency. The replicated standard deviations also encompassed the observed value ( 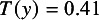 ). The predictive distributions of the standard deviation were somewhat wider than under the random-effects model, especially for larger *K*. This pattern reflects the additional variability introduced by explicitly modeling bias and is consistent with the model’s design to partition total variability into heterogeneity and bias components.

Overall, the posterior predictive checks confirm that both modeling approaches adequately represent the data. The random-effects model provides a tighter predictive fit, while the bias-adjustment models introduce greater flexibility to account for uncertainty in risk-of-bias status. This trade-off, seen in slightly wider predictive distributions, ensures that inferences remain robust to potential systematic biases across studies. These evaluation results provide important context for the subsequent model comparison using WAIC. Whereas posterior predictive checks assess whether models can reproduce the observed data, WAIC formally balances model fit against complexity to determine predictive performance. Together, these complementary approaches allow us to distinguish between models that merely fit the data well and those that provide the most reliable generalization beyond the observed studies.

### 6.4 Results of model comparison

Model comparison using WAIC revealed clear differences in predictive performance between the random-effects and bias-adjustment models as presented in Table 2. The random-effects model achieved the lowest WAIC (2.38), indicating superior overall predictive accuracy relative to the bias-adjustment models, whose WAIC values ranged from 8.64 ( 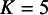 ) to 9.25 ( 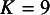 ). This advantage reflects the balance between model fit and complexity: although the bias-adjustment models exhibited slightly higher log pointwise predictive densities (LPPD = 33.52–33.68) than the random-effects model (LPPD = 33.42), they incurred larger effective parameter penalties (pWAIC = 37.84–38.15 vs. 34.61). Thus, the additional flexibility of modeling bias improved fit only marginally, while substantially increasing complexity, leading to worse predictive performance under WAIC.

**Table 2:** Model comparison using WAIC criteria between random-effects and bias-adjustment models

Model	WAIC	LPPD	pWAIC
Random effects	2.38	33.42	34.61
Bias adjustment ( 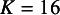 )	8.73	33.68	38.05
Bias adjustment ( 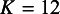 )	8.92	33.55	38.01
Bias adjustment ( 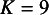 )	9.25	33.53	38.15
Bias adjustment ( 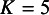 )	8.64	33.52	37.84

*Note*: WAIC = Watanabe–Akaike information criterion; LPPD = log pointwise predictive density; pWAIC = effective number of parameters.

Within the bias-adjustment models, the 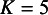 specification provided the most favorable trade-off, achieving the lowest WAIC among bias-adjusted variants (8.64). This configuration balanced relatively modest complexity (pWAIC = 37.84) with fit comparable to other specifications, suggesting that more extreme prior informativeness did not yield practical gains in predictive accuracy. By contrast, the 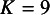 model performed worst (WAIC = 9.25), primarily due to its higher complexity (pWAIC = 38.15). Although the WAIC differences among bias-adjustment models were small ( 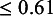 ), they consistently indicate that stronger priors on bias prevalence improved efficiency without altering model fit substantially.

Figure 3 presents the overall effect size estimates and 95% credible intervals. The random-effects model produced an estimated mean effect of approximately 0.12 with a relatively narrow credible interval, consistent with its tighter posterior predictive performance. The bias-adjustment models, in contrast, displayed systematically smaller overall effect sizes, with stronger shrinkage toward zero as *K* decreased from 16 to 5. This pattern reflects the increasing weight assigned to potential bias, leading to more conservative estimates when stronger prior information is imposed. Importantly, all bias-adjusted estimates exhibited wider credible intervals than the random-effects estimate, particularly for larger *K*, highlighting the trade-off between accounting for bias and inflating uncertainty.

Within the bias-adjustment framework, the decomposition into biased and unbiased effect size estimates further illustrates this trade-off. Across all *K* values, unbiased estimates consistently shifted downward relative to biased ones, indicating that the adjustment primarily operated by correcting for a positive bias component. However, these unbiased intervals were also wider than those for biased effects, underscoring the additional uncertainty introduced by the bias-adjustment process. These findings present a potential conflict between statistical model selection criteria and the substantive goals of the analysis. While a simpler random-effects model may demonstrate superior predictive performance according to metrics (WAIC), a bias-adjustment model is arguably more theoretically defensible when an evidence base is characterized by a high risk of bias. In such contexts, the primary analytical objective is not necessarily to maximize predictive accuracy, but rather to derive an effect estimate that has been corrected for known methodological flaws, even at the cost of reduced precision. Therefore, while theoretical grounds can warrant selecting a bias-adjustment model over a random-effects model, criteria should then be used to identify the optimal specification among the set of candidate bias-adjustment models.

### 6.5 Results of simulation data

To evaluate the bias-adjustment model’s performance under known conditions, we conducted a simulation study using 100 studies with effect size and risk-of-bias characteristics patterned after the empirical dataset (approximately 3% low, 43% unclear, and 54% high risk). The data-generating process specified true parameter values of 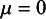 for the unbiased effect, 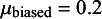 for the biased effect, 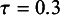 for heterogeneity, 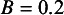 for bias magnitude, and 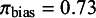 for the bias prevalence. Bias-adjustment models were then fitted using four prior specifications for 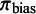 , corresponding to 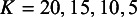 and derived from targeted median values. These priors reflected progressively stronger information about bias prevalence: ( 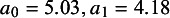 ) for 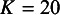 , ( 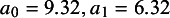 ) for 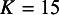 , ( 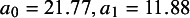 ) for 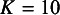 , and ( 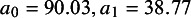 ) for 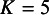 . Posterior estimates, convergence diagnostics ( 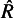 ), and discrepancies from the true parameter values are summarized in Table 3, with forest plots shown in Figure 4.

**Table 3:** Posterior summaries for bias-adjustment model across sensitivity analyses in simulation data

Parameter	Mean	SD	95% CrI	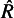	Discrepancy
*Bias-adjustment model* ( 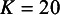 )					
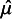	0.01	0.11	[ 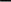 0.22, 0.17]	1.02	0.01
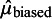	0.26	0.11	[0.08, 0.52]	1.00	0.06
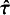	0.26	0.06	[0.16, 0.38]	1.00	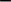 0.04
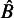	0.24	0.16	[0.01, 0.58]	1.00	0.04
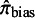	0.61	0.13	[0.35, 0.86]	1.00	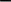 0.12
*Bias-adjustment model* ( 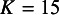 )					
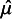	0.01	0.10	[ 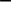 0.20, 0.17]	1.00	0.01
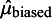	0.25	0.10	[0.08, 0.49]	1.00	0.05
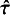	0.25	0.05	[0.16, 0.36]	1.00	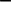 0.05
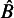	0.24	0.15	[0.01, 0.55]	1.00	0.04
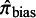	0.62	0.11	[0.40, 0.83]	1.00	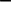 0.11
*Bias-adjustment model* ( 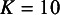 )					
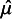	0.01	0.09	[ 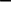 0.20, 0.17]	1.00	0.01
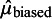	0.24	0.09	[0.08, 0.44]	1.00	0.04
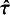	0.24	0.05	[0.16, 0.34]	1.00	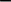 0.06
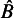	0.23	0.14	[0.01, 0.53]	1.00	0.03
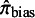	0.65	0.08	[0.49, 0.80]	1.00	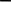 0.08
*Bias-adjustment model* ( 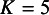 )					
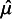	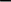 0.01	0.10	[ 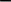 0.21, 0.16]	1.00	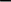 0.01
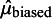	0.22	0.08	[0.08, 0.39]	1.00	0.02
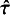	0.23	0.04	[0.15, 0.32]	1.00	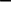 0.07
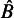	0.23	0.14	[0.01, 0.52]	1.00	0.03
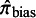	0.70	0.04	[0.62, 0.78]	1.00	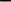 0.03

*Note*: 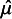 = unbiased mean effect; 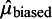 = biased mean effect; 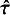 = heterogeneity standard deviation; 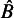 = bias magnitude; 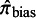 = (posterior) probability of bias; 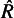 = potential scale reduction factor; SD = standard deviation; CrI = credible interval; Discrepancy = difference between the posterior mean estimate and the true value ( 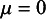 , 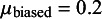 , 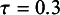 , 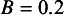 , 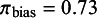 ).

Across all prior settings, the models accurately recovered the true overall effect size. Discrepancies for 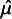 were negligible ( 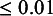 ), and all 95% credible intervals included the true value of 0. The biased effect size 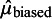 also closely approximated the true value of 0.2, with discrepancies declining as prior informativeness increased (from 0.06 at 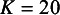 to 0.02 at 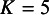 ). This pattern reflects the model’s improved ability to isolate the bias component when stronger prior information is provided. Heterogeneity estimates were consistently underestimated, with 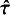 discrepancies ranging from 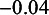 ( 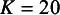 ) to 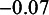 ( 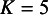 ), although all credible intervals encompassed the true 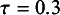 . The reduction in 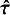 with decreasing *K* mirrors trends observed in the empirical analysis, suggesting that bias adjustment partially reallocates variability from heterogeneity to systematic bias.

Bias magnitude estimates 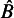 were stable across conditions, with discrepancies of 0.03–0.04 and credible intervals consistently covering the true value of 0.2. Estimates of bias prevalence 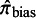 were more sensitive to prior specification: higher *K* values yielded underestimation (e.g., 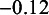 at 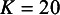 ), while only the 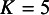 model produced a credible interval containing the true 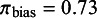 . Among the four specifications, 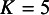 yielded the smallest discrepancies across all parameters, with credible intervals consistently covering the true values while appropriately reflecting posterior uncertainty. This suggests that more informative priors for bias prevalence enhance estimation accuracy without over-constraining the model. Figure 4 illustrates these trends: 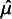 estimates cluster tightly around zero across all *K*, while 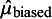 converges toward 0.2 as prior informativeness increases. The consistency between these simulation results and the empirical findings strengthens confidence in the bias-adjustment model’s validity and the robustness of the Bayesian workflow applied in this study. By demonstrating accurate recovery of known parameters under realistic conditions, the simulation provides critical evidence that the workflow and modeling choices support reliable inference in applied evidence synthesis contexts.

## 7 Implications and conclusions

This study contributes to the advancement of meta-analytic methodology by demonstrating how a Bayesian workflow can strengthen bias adjustment in evidence synthesis. A key aspect of this contribution is the extended bias-adjustment model employed within our workflow, which, unlike existing Bayesian models, is designed to directly incorporate the three levels (low, unclear, and high) common in risk-of-bias assessments. Its primary advantage is the ability to statistically account for the influence of studies with an “unclear” risk of bias, the magnitude of which can be flexibly controlled by a specific model parameter. By integrating prior predictive checks, sensitivity analysis, model fitting, posterior predictive checks, model comparison, and simulation-based validation, we show how the workflow provides a systematic approach to evaluating the robustness of bias-adjusted estimates.

The findings highlight several insights. Prior predictive checks revealed that incorporating bias adjustment widened the range of plausible effect sizes relative to the random-effects model, reflecting the additional uncertainty that arises when study rigorousness is explicitly modeled. Posterior predictive checks confirmed that both random-effects and bias-adjustment models reproduced the observed distributional features of the data, while the bias-adjustment models produced broader replicated distributions, consistent with the expectation that modeling bias increases variability. Sensitivity analysis further demonstrated that the unbiased mean effect estimates were responsive to different prior specifications on bias prevalence, with stronger priors shifting estimates closer to the null and reducing residual heterogeneity.

This highlights both the influence of prior assumptions and the importance of systematically testing their impact within the workflow. Model comparison using WAIC indicated that the simpler random-effects model provided better predictive accuracy overall; however, within the bias-adjustment framework, models with stronger prior information on bias prevalence performed more stably and achieved a more favorable balance between fit and complexity. Importantly, the overall effect size estimates demonstrated that bias adjustment systematically reduced estimated effects while widening credible intervals, providing evidence that the models appropriately accounted for potential upward bias in the primary studies. The simulation study further validated these findings by demonstrating that the bias-adjustment models were capable of recovering true parameter values under conditions that mimicked the empirical dataset.

Despite these strengths, several limitations warrant consideration. Estimates of heterogeneity were consistently attenuated in both real and simulated analyses, suggesting that some variability was absorbed into the bias parameters and highlighting potential challenges in disentangling sources of variance. Moreover, the sensitivity of results to the prior specification for bias prevalence illustrates the weak identifiability of this parameter and emphasizes the importance of grounding prior choices in domain knowledge or empirical calibration.^13^ The generalizability of our findings is also limited by reliance on a single dataset with a relatively high proportion of studies at elevated risk of bias; applications to more heterogeneous evidence bases are needed to fully evaluate the model’s utility.

The challenge of specifying these priors is a central issue in the Bayesian meta-analysis, and expert elicitation offers an approach to formally translate domain knowledge into quantitative prior distributions. Foundational work in this area demonstrated how elicited opinions could be used to construct study-specific priors that formally down-weight less rigorous or relevant evidence; such adjustments not only shift the combined estimate but also substantially increase its variance, a finding consistent with our own results.^8^ More recently, methods have been developed to blend expert judgment with empirical data, anchoring these priors more robustly. This hybrid approach combines expert opinion on specific trials with empirical bias distributions derived from large collections of existing meta-analyses, thereby leveraging both context-specific and broad evidence to develop the informative priors our simulation study found most effective.^9^

In summary, the Bayesian workflow provides a principled framework for conducting a credible bias-adjusted meta-analysis. This systematic approach ensures that researchers to be transparent about their assumptions, test them rigorously, and present a more complete picture of the uncertainty surrounding an effect size estimate. By demonstrating how this process enhances the reliability and interpretability of bias-adjusted models, we aim to promote the broader adoption of these powerful techniques in contexts where bias threatens the validity of evidence synthesis.

## Supporting information

Jung and Aloe supplementary material Jung and Aloe supplementary material

## Figures and Tables

**Figure A2:**
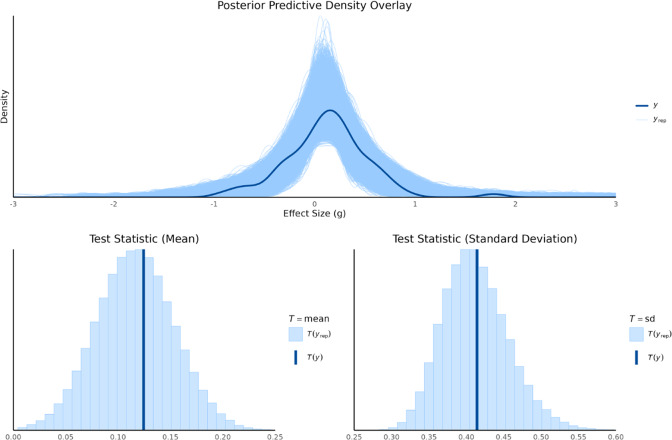
Posterior predictive density overlay and test statistics for bias-adjustment model ( 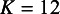 ).

**Figure A3:**
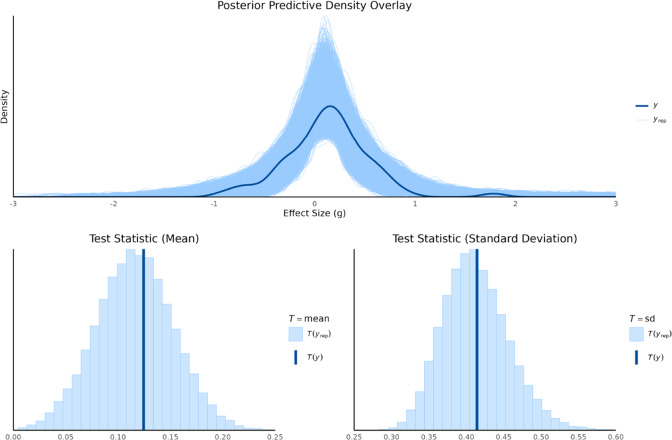
Posterior predictive density overlay and test statistics for bias-adjustment model ( 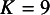 ).

## Data Availability

The datasets used and analyzed in this study are available in the public repository at https://osf.io/fby7w/. A subset of the data can be generated using the code included in the Supplementary Material.

## Supplementary material

To view supplementary material for this article, please visit https://doi.org/10.1017/rsm.2025.10050.

## Appendix: Figures

This appendix presents a concise overview of the sensitivity analyses for the bias-adjustment model. We explored how posterior estimates for the unbiased mean (  ), biased mean (  ), heterogeneity (  ), bias magnitude (  ), and the probability of bias (  ) change as the prior becomes more informative by decreasing the parameter *K*.
