# Supplementary material for: Bayesian workflow for bias-adjustment model in meta-analysis
Source: Res Synth Methods. 2025 Nov 13;17(2):293–313. doi: 10.1017/rsm.2025.10050 (PMC12873618; doi:10.1017/rsm.2025.10050)
Supplement: Jung and Aloe supplementary material [file S1759287925100501sup001.pdf]

# Bayesian Workflow for Bias Adjustment Model in Meta-Analysis

Juyoung Jung and Ariel M. Aloe

October 1, 2025

## Contents

**1 Introduction** **1**

**2 Real Data Analysis** **2**

2.1 Data Manipulation . . . . . . . . 2
2.2 Model Specification . . . . . . . . 2
2.3 Prior Predictive Checking . . . . . . . . 3
2.4 Model Fitting (Random Effects) . . . . . . . . 7
2.5 Model Fitting (Bias Adjustment) . . . . . . . . 8
2.6 Model Comparison (WAIC) . . . . . . . . 12
2.7 Posterior Distribution Plots . . . . . . . . 14
2.8 Posterior Predictive Checking . . . . . . . . 16
2.9 Reporting Results and Visualization . . . . . . . . 17

**3 Simulation Data Analysis** **21**

# 1 Introduction

This supplemental material contains the R code used to conduct the analyses in *Bayesian Workflow for Bias Adjustment Model in Meta-Analysis.* Each code chunk is accompanied by a brief explanation of its function.

The following R packages are required for the analysis:

```
required_packages <- c("dplyr", "R2jags", "ggplot2", "LaplacesDemon",
                       "tidyr", "devEMF", "LearnBayes", "coda", "tibble", "grid",
                       "gridExtra", "bayesplot", "patchwork")
for (package in required_packages) {
  if (!require(package, character.only = TRUE)) {
    install.packages(package)
    library(package, character.only = TRUE)
  }
}
```

Set a seed for reproducibility where applicable:

```
set.seed(2025)
```

# 2 Real Data Analysis

Data is sourced from an RDS file (`coteach_data.rds`) and paths are defined for output and BUGS model files.

```
coteach_dat <- readRDS("~/Bayesian Workflow/BABW/coteach_data.rds")
```

## 2.1 Data Manipulation

The dataset is filtered and transformed. 1) Filtering: Excludes follow-up studies and specific study-year combinations. 2) Recoding: Simplifies subject categories (e.g., "Language arts" to "LA"). 3) Indicator Creation: Assigns binary indicators for risk of bias based on the overall variable. 4) Subsetting: Selects one effect size per study to avoid dependency.

```
dat <- coteach_dat %>%
  mutate(subject = recode(subject, "Language arts" = "LA")) %>%
  filter(follow_up == "No") %>%
  filter(!(study_year == "Schaef 2014" & population_type == "Special needs students")) %>%
  dplyr::select(study, design, g, vg, overall = Overall) %>%
  mutate(
    indicator = case_when(
      overall == "Low" ~ 0,
      overall %in% c("Moderate", "Some concerns") ~ rbinom(n(), size = 1, prob = 0.5),
      overall %in% c("Serious", "High") ~ 1,
      TRUE ~ NA_real_
    ),
    overall = case_when(
      overall %in% c("Moderate", "Some concerns") ~ "Unclear",
      overall %in% c("Serious", "High") ~ "High",
      TRUE ~ overall
    )
  ) %>%
  filter(!is.na(vg) & vg > 0 & !is.na(g)) %>%
  mutate(se.y = sqrt(vg)) %>%
  group_by(study) %>%
  sample_n(1) %>%
  ungroup()
```

## 2.2 Model Specification

Two Bayesian models (Bayesian random effect model and Bayesian bias adjustment model) are specified in BUGS syntax:

```
# Bayesian random effect model
random_effects_model <- "
model {
  for(i in 1:N) {
    # Likelihood
    y[i] ~ dnorm(theta[i], pre.y[i])
    pre.y[i] <- pow(se.y[i], -2)

    # Process model
    theta[i] ~ dnorm(mu, prec.tau)

    # Posterior predictive simulation
    y.rep[i] ~ dnorm(theta[i], pre.y[i])
  }

  # Priors for hyperparameters
```

```
  tau <- 1 / sqrt(prec.tau)
  prec.tau ~ dscaled.gamma(scale.sigma.between, df.scale.between)
  mu ~ dnorm(0, 0.1)

  # Posterior predictive distribution
  theta.new ~ dnorm(mu, prec.tau)
}"
writeLines(random_effects_model, bugs_re_path)

# Bayesian bias adjustment model
bias_adjustment_model <- "
model {
  for(i in 1:N) {
    # Likelihood
    y[i] ~ dnorm(theta.ba[i], pre.y[i])
    pre.y[i] <- pow(se.y[i], -2)

    # Process model accounting for bias
    theta.ba[i] <- theta[i]*(1 - I[i]) + theta.bias[i]*I[i]
    I[i] <- T[i] - 1

    theta[i] ~ dnorm(mu[1], prec.tau[i])
    theta.bias[i] ~ dnorm(mu[2], prec.tau[i])

    T[i] ~ dcat(p.bias[1:2])

    # Precision calculation
    prec.tau[i] <- inv.var[T[i]] * w[T[i], i]
    w[1,i] <- 1
    w[2,i] ~ dbeta(nu, 1)

    # Posterior predictive simulation
    y.rep[i] ~ dnorm(theta.ba[i], pre.y[i])
  }

  # Priors and definitions
  nu <- 1/2

  p.bias[2] ~ dbeta(a0, a1)
  p.bias[1] <- 1 - p.bias[2]

  tau <- 1 / sqrt(inv.var[1])
  inv.var[1] ~ dscaled.gamma(scale.sigma.between, df.scale.between)
  inv.var[2] <- inv.var[1]

  mu[1] ~ dnorm(0, 1)
  B ~ dunif(0, B.max)
  mu[2] <- mu[1] + B
}"
writeLines(bias_adjustment_model, bugs_ba_path)
```

## 2.3 Prior Predictive Checking

Prior predictive simulations are conducted to evaluate the appropriateness of priors by generating simulated effect sizes under both models.

```
## Simulation setup with parameter values (aligned with BUGS models and Verde's BC model)
n_sim <- 10000
N <- nrow(dat)
scale.sigma.between <- 0.3; df.scale.between <- 1  # tau ~ dscaled.gamma(0.5, 1)
mu_mean <- 0.0; mu_prec <- 0.1                     # mu ~ dnorm(0, 0.1) from BUGS
a0 <- 1; a1 <- 1                                   # p.bias[2] ~ dbeta(1, 1)
```

```r
B.max <- 2.5                                              # B ~ dunif(0, B.max)
nu <- 0.5                                                 # nu fixed at 1/2 as in BUGS
hypothetical_se.y <- dat$se.y                             # se.y directly from the data
hypothetical_pre.y <- 1 / (hypothetical_se.y^2)    # calculate precision as in BUGS

## Storage for simulated data
# Random effects model
sim_results_re <- list(
  mu = numeric(n_sim),
  tau = numeric(n_sim),
  theta = matrix(NA, nrow = n_sim, ncol = N),
  y_rep = matrix(NA, nrow = n_sim, ncol = N)
)
# Bias adjustment model
sim_results_ba <- list(
  mu1 = numeric(n_sim),                        # mu[1] (unbiased mean)
  mu2 = numeric(n_sim),                        # mu[2] (biased mean)
  B = numeric(n_sim),                          # B (bias shift)
  tau = numeric(n_sim),                        # between-study variability
  p_bias2 = numeric(n_sim),                    # p.bias[2] (probability of bias)
  T_i = matrix(NA, nrow = n_sim, ncol = N), # bias indicator T[i]
  theta_ba = matrix(NA, nrow = n_sim, ncol = N),
  y_rep = matrix(NA, nrow = n_sim, ncol = N)
)

for (s in 1:n_sim) {

  ### For Bayesian random effects model simulation
  ## 1. Simulate hyperparameters from priors
  sim_mu_re <- rnorm(1, mean = mu_mean, sd = 1 / sqrt(mu_prec))
  sim_prec.tau_re <- rgamma(1, shape = df.scale.between / 2,  # prec.tau ~ dscaled.gamma
                          rate = df.scale.between * scale.sigma.between^2 / 2)
  if (sim_prec.tau_re <= 0) sim_prec.tau_re <- 1e-6
  sim_tau_re <- 1 / sqrt(sim_prec.tau_re)
  sim_results_re$mu[s] <- sim_mu_re
  sim_results_re$tau[s] <- sim_tau_re

  ## 2. Simulate study-specific true effects
  sim_theta_i_re <- rnorm(N, mean = sim_mu_re, sd = sim_tau_re)
  sim_results_re$theta[s, ] <- sim_theta_i_re

  ## 3. Simulate observed effect sizes
  sim_y_i_re <- rnorm(N, mean = sim_theta_i_re, sd = hypothetical_se.y)
  sim_results_re$y_rep[s, ] <- sim_y_i_re

  ### For Bayesian bias adjustment model simulation (with slash distribution)
  ## 1. Simulate hyperparameters from priors
  sim_mu1_ba <- rnorm(1, mean = mu_mean, sd = 1 / sqrt(mu_prec))  # mu[1] ~ dnorm(0, 0.1)
  sim_B_ba <- runif(1, min = 0, max = B.max)                     # B ~ dunif(0, B.max)
  sim_mu2_ba <- sim_mu1_ba + sim_B_ba                            # mu[2] = mu[1] + B
  sim_p_bias2_ba <- rbeta(1, shape1 = a0, shape2 = a1)           # p.bias[2] ~ dbeta(a0, a1)
  sim_p_bias_ba <- c(1 - sim_p_bias2_ba, sim_p_bias2_ba)         # p.bias[1] = 1 - p.bias[2]
  sim_inv.var1_ba <- rgamma(1, shape = df.scale.between / 2,     # inv.var[1] ~ dscaled.gamma
                          rate = df.scale.between * scale.sigma.between^2 / 2)
  if (sim_inv.var1_ba <= 0) sim_inv.var1_ba <- 1e-6
  sim_tau_ba <- 1 / sqrt(sim_inv.var1_ba)
  sim_results_ba$mu1[s] <- sim_mu1_ba
  sim_results_ba$B[s] <- sim_B_ba
  sim_results_ba$mu2[s] <- sim_mu2_ba
  sim_results_ba$tau[s] <- sim_tau_ba
  sim_results_ba$p_bias2[s] <- sim_p_bias2_ba

  ## 2. Simulate study-specific parameters and true effects
  sim_theta_ba_i <- numeric(N)
```

```
  sim_T_i <- numeric(N)
  for (i in 1:N) {
    # Simulate bias status T[i] (1 = unbiased, 2 = biased)
    sim_T_i[i] <- sample(1:2, size = 1, prob = sim_p_bias_ba)
    sim_I_i <- sim_T_i[i] - 1

    # Simulate weight w[2,i] for biased studies
    sim_w_i <- ifelse(sim_T_i[i] == 1, 1, rbeta(1, nu, 1))

    # Precision calculation
    sim_prec.tau_i <- sim_inv.var1_ba * sim_w_i
    if (sim_prec.tau_i <= 0) sim_prec.tau_i <- 1e-6
    sim_sd_tau_i <- 1 / sqrt(sim_prec.tau_i)

    # Simulate theta[i] and theta.bias[i]
    sim_theta_i <- rnorm(1, mean = sim_mu1_ba, sd = sim_tau_ba)
    if (sim_T_i[i] == 1) {
      sim_theta_ba_i[i] <- sim_theta_i
    } else {
      z <- rnorm(1, mean = 0, sd = sim_sd_tau_i)
      u <- runif(1, 0, 1)
      sim_theta_ba_i[i] <- sim_mu2_ba + z / u
    }
  }
  sim_results_ba$T_i[s, ] <- sim_T_i
  sim_results_ba$theta_ba[s, ] <- sim_theta_ba_i

  ## 3. Simulate observed effect sizes
  sim_y_i_ba <- rnorm(N, mean = sim_theta_ba_i, sd = hypothetical_se.y)
  sim_results_ba$y_rep[s, ] <- sim_y_i_ba

}

y_rep_re_long <- as.data.frame(sim_results_re$y_rep) %>%
  mutate(sim = 1:n_sim) %>%
  pivot_longer(cols = -sim, names_to = "study_rep", values_to = "y_rep") %>%
  mutate(model = "Random Effects")

y_rep_ba_long <- as.data.frame(sim_results_ba$y_rep) %>%
  mutate(sim = 1:n_sim) %>%
  pivot_longer(cols = -sim, names_to = "study_rep", values_to = "y_rep") %>%
  mutate(model = "Bias Adjustment")

y_rep_combined <- bind_rows(y_rep_re_long, y_rep_ba_long) %>%
  mutate(
    model = recode(model,
                   "Random Effects" = "Random Effect Model",
                   "Bias Adjustment" = "Bias Adjustment Model"),
    model = factor(model, levels = c("Random Effect Model", "Bias Adjustment Model"))
  )

# Calculate mean values for each model
mean_values <- y_rep_combined %>%
  group_by(model) %>%
  summarise(mean_y_rep = mean(y_rep, na.rm = TRUE)) %>%
  ungroup()

# Observed mean from dat$g
obs_mean <- mean(dat$g, na.rm = TRUE)

# Create labels for annotate
label_re <- sprintf("mu[RE] == %.2f",
                    round(mean_values$mean_y_rep[mean_values$model == "Random Effect Model"], 2))
label_ba <- sprintf("mu[BC] == %.2f",
```

```
                    round(mean_values$mean_y_rep[mean_values$model == "Bias-Corrected Model"], 2))
label_obs <- sprintf("Observed~Mean~(bar(g))~'='~%.2f", obs_mean)

# Plot with observed mean overlay and text
plot_prior_pred <- ggplot(y_rep_combined, aes(x = y_rep, fill = model, color = model)) +
  stat_density(geom = "area", alpha = 0.5, position = "identity", trim = FALSE, bw = "nrd0") +
  scale_fill_manual(values = c("Random Effect Model" = "orange",
                               "Bias Adjustment Model" = "lightblue")) +
  scale_color_manual(values = c("Random Effect Model" = "darkorange",
                                "Bias Adjustment Model" = "blue")) +
  scale_x_continuous(limits = c(-30, 30), breaks = seq(-20, 20, by = 10), expand = c(0, 0)) +
  scale_y_continuous(limits = c(0, 0.12), breaks = seq(0, 0.12, by = 0.03), expand = c(0, 0)) +
  geom_vline(xintercept = obs_mean, color = "red", linetype = "solid", linewidth = 0.7) +
  annotate("text", x = obs_mean, y = Inf, label = label_obs, parse = TRUE,
           color = "red", size = 11 / .pt, hjust = -0.1, vjust = 1.5) +
  labs(x = expression("Simulated Effect Size " ~ (y^{rep})),
       y = "Density", fill = "Model", color = "Model") +
  theme_minimal(base_size = 11) +
  theme(
    legend.position = "bottom",
    panel.grid.major = element_blank(),
    panel.grid.minor = element_blank(),
    axis.text = element_text(size = 11),
    axis.title = element_text(size = 11),
    legend.title = element_text(size = 11),
    legend.text = element_text(size = 11),
    axis.line = element_line(color = "black", linewidth = 0.3),
    plot.margin = margin(10, 10, 10, 10)
  )

# Print the plot
print(plot_prior_pred)
```

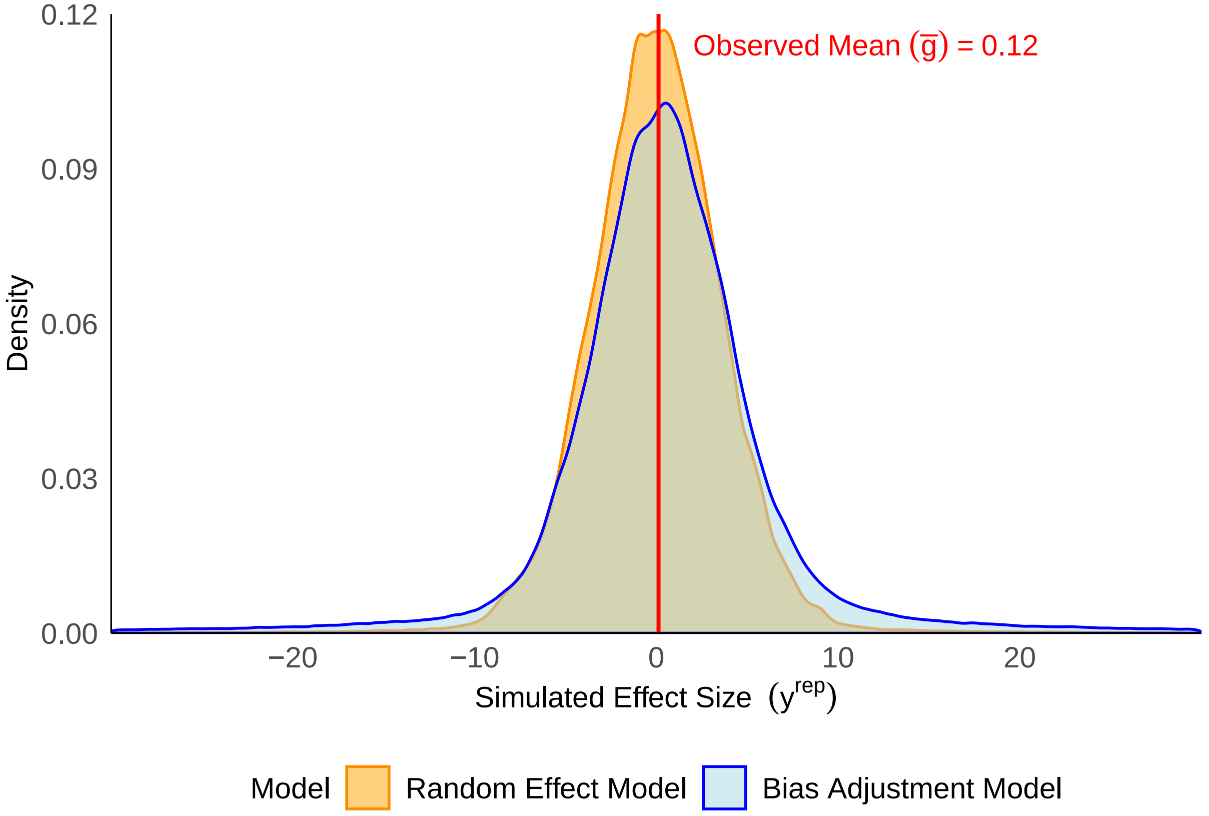


## 2.4 Model Fitting (Random Effects)

The random effects model is fitted using JAGS with 4 chains, 200,000 iterations, and a burn-in of 40,000. Posterior summaries are saved.

```
# Define invlogit function
invlogit <- function(x) 1 / (1 + exp(-x))

# Prepare data list
data_re <- list(
  N = N,
  y = dat$g,
  se.y = dat$se.y,
  scale.sigma.between = 0.3,
  df.scale.between = 1
)

# Parameters to monitor
params_re <- c("mu", "tau", "theta", "theta.new", "y.rep")

jags_re <- jags(
  data = data_re,
  inits = NULL,
  parameters.to.save = params_re,
  model.file = bugs_re_path,
  n.chains = 4,
  n.iter = 200000,
  n.burnin = 40000,
  n.thin = 10,
```

```
  quiet = FALSE
)
```

```
## Compiling model graph
##    Resolving undeclared variables
##    Allocating nodes
## Graph information:
##    Observed stochastic nodes: 76
##    Unobserved stochastic nodes: 155
##    Total graph size: 393
##
## Initializing model
```

```
# Extract and summarize posterior results
posterior_re_summary <- jags_re$BUGSoutput$summary

# Create a data frame with posterior summaries and R-hat
param_rows_re <- !grepl("^y\\.rep\\[", rownames(posterior_re_summary)) &
  !grepl("^theta\\[", rownames(posterior_re_summary))
results_re_df <- data.frame(
  Parameter = rownames(posterior_re_summary)[param_rows_re],
  Mean = posterior_re_summary[param_rows_re, "mean"],
  SD = posterior_re_summary[param_rows_re, "sd"],
  CI_Lower_95 = posterior_re_summary[param_rows_re, "2.5%"],
  Median = posterior_re_summary[param_rows_re, "50%"],
  CI_Upper_95 = posterior_re_summary[param_rows_re, "97.5%"],
  Rhat = posterior_re_summary[param_rows_re, "Rhat"],
  row.names = NULL
)

# Print the results
print(results_re_df)
```

```
##   Parameter        Mean          SD  CI_Lower_95      Median CI_Upper_95
## 1  deviance -44.3232064 12.21011406 -66.54671296 -44.8850283 -18.6621606
## 2        mu   0.1161917  0.04334869   0.03128672   0.1160204   0.2016938
## 3       tau   0.3142797  0.03638243   0.24964271   0.3119650   0.3915980
## 4 theta.new   0.1151048  0.31842303  -0.51057700   0.1133191   0.7413601
##       Rhat
## 1 1.000995
## 2 1.001006
## 3 1.000978
## 4 1.000981
```

## 2.5 Model Fitting (Bias Adjustment)

The bias adjustment model is fitted with sensitivity analysis over different `K` values, adjusting the Beta prior for bias probability.

```
# Function to calculate K based on median bias probability
calculate_K <- function(median, total_studies, high_risk_studies) {
  K <- high_risk_studies - median * total_studies
  return(round(K))
}

# Function to calculate Beta parameters (a0 and a1) for a given K
calculate_beta_params <- function(K, n_rob, n_effect) {
  x1 <- (n_rob - K) / n_effect        # 50th percentile (median)
  x2 <- n_rob / n_effect              # 90th percentile
  quantile1 <- list(p = 0.5, x = x1)
  quantile2 <- list(p = 0.9, x = x2)
```

```r
  # Ensure percentiles are valid (0 < x < 1)
  if (x1 <= 0 || x1 >= 1 || x2 <= 0 || x2 >= 1 || x1 >= x2) {
    warning(paste("Invalid percentiles for K =", K, "(x1 =", round(x1,3),
                  ", x2 =", round(x2,3), "). Cannot calculate Beta params."))
    return(c(a0 = NA, a1 = NA))
  }

  beta_par <- tryCatch({
    LearnBayes::beta.select(quantile1, quantile2)
  }, error = function(e) {
    warning(paste("Error in beta.select for K =", K, ":", e$message))
    return(c(NA, NA))
  })
  return(c(a0 = beta_par[1], a1 = beta_par[2]))
}

# Prepare data
n_rob <- sum(dat$indicator == 1, na.rm = TRUE)
cat("Number of high-risk studies (n_rob):", n_rob, "\n")
```

```
## Number of high-risk studies (n_rob): 58
```

```r
# Calculate Beta parameters for median
target_medians <- c(0.55, 0.60, 0.65, 0.70)
beta_param <- data.frame(Median = target_medians) %>%
  mutate(
    K = sapply(Median, calculate_K, total_studies = N, high_risk_studies = n_rob),
    Beta_Params = lapply(K, calculate_beta_params, n_rob = n_rob, n_effect = N),
    a0 = sapply(Beta_Params, `[[`, 1),
    a1 = sapply(Beta_Params, `[[`, 2)
  ) %>%
  select(Median, K, a0, a1) %>%
  filter(!is.na(a0))

print(beta_param)
```

```
##   Median  K    a0    a1
## 1   0.55 16  4.50  3.70
## 2   0.60 12  8.31  5.54
## 3   0.65  9 15.19  8.52
## 4   0.70  5 50.81 22.24
```

```r
# Create storage for results, posterior samples, and JAGS objects
results_ba <- list()
posterior_ba <- list()
jags_ba_list <- list()

# Define index of biased study T
T_init <- rep(NA, N)

# Parameters to monitor - ADD y.rep
params_ba <- c("mu", "tau", "theta", "theta.ba", "theta.bias", "p.bias", "I", "w", "B", "y.rep")

# Sensitivity analysis across valid K values
K_values <- beta_param$K
k_labels_vec <- paste0("K_", K_values)

if(length(K_values) == 0) {
  stop("No valid K values found for sensitivity analysis. Check calculations.")
}
```

```
for (k_idx in seq_along(K_values)) {
  K <- K_values[k_idx]
  k_label <- k_labels_vec[k_idx]
  a0 <- beta_param$a0[k_idx]
  a1 <- beta_param$a1[k_idx]

  # Data list for bias adjustment model
  data_ba <- list(
    N = N,
    y = dat$g,
    se.y = dat$se.y,
    scale.sigma.between = 0.3,
    df.scale.between = 1,
    a0 = a0,
    a1 = a1,
    B.max = 10
  )

  # Fit model
  jags_ba <- jags(
    data = data_ba,
    inits = NULL,
    parameters.to.save = params_ba,
    model.file = bugs_ba_path,
    n.chains = 4,
    n.iter = 200000,
    n.burnin = 40000,
    n.thin = 10,
    quiet = FALSE
  )

  # Store JAGS object
  jags_ba_list[[k_label]] <- jags_ba

  # Extract and summarize posterior results
  posterior_ba_summary <- jags_ba$BUGSoutput$summary

  # Create a data frame with posterior summaries and R-hat
  param_rows_ba <- !grepl("^y\\.rep\\[", rownames(posterior_ba_summary)) &
    !grepl("^(theta|theta\\.ba|theta\\.bias)\\[", rownames(posterior_ba_summary)) &
    !grepl("^(I|T|w)\\[", rownames(posterior_ba_summary))

  results_ba[[k_label]] <- data.frame(
    Parameter = rownames(posterior_ba_summary)[param_rows_ba],
    Mean = posterior_ba_summary[param_rows_ba, "mean"],
    SD = posterior_ba_summary[param_rows_ba, "sd"],
    CI_Lower_95 = posterior_ba_summary[param_rows_ba, "2.5%"],
    Median = posterior_ba_summary[param_rows_ba, "50%"],
    CI_Upper_95 = posterior_ba_summary[param_rows_ba, "97.5%"],
    Rhat = posterior_ba_summary[param_rows_ba, "Rhat"],
    row.names = NULL
  )

  # Store posterior samples for key parameters
  posterior_ba[[k_label]] <- data.frame(
    K = K,
    mu1 = jags_ba$BUGSoutput$sims.list$mu[, 1],
    mu2 = jags_ba$BUGSoutput$sims.list$mu[, 2],
    tau = jags_ba$BUGSoutput$sims.list$tau,
    B = jags_ba$BUGSoutput$sims.list$B,
    p.bias2 = jags_ba$BUGSoutput$sims.list$p.bias[, 2]
  )

}
```

```
## Compiling model graph
##    Resolving undeclared variables
##    Allocating nodes
## Graph information:
##    Observed stochastic nodes: 76
##    Unobserved stochastic nodes: 384
##    Total graph size: 1160
##
## Initializing model
##
## Compiling model graph
##    Resolving undeclared variables
##    Allocating nodes
## Graph information:
##    Observed stochastic nodes: 76
##    Unobserved stochastic nodes: 384
##    Total graph size: 1160
##
## Initializing model
##
## Compiling model graph
##    Resolving undeclared variables
##    Allocating nodes
## Graph information:
##    Observed stochastic nodes: 76
##    Unobserved stochastic nodes: 384
##    Total graph size: 1160
##
## Initializing model
##
## Compiling model graph
##    Resolving undeclared variables
##    Allocating nodes
## Graph information:
##    Observed stochastic nodes: 76
##    Unobserved stochastic nodes: 384
##    Total graph size: 1160
##
## Initializing model
```

```
# Print the results
print(results_ba)
```

```
## $K_16
##   Parameter         Mean          SD   CI_Lower_95       Median CI_Upper_95
## 1         B   0.20524525  0.15620623   0.008310418   0.17606322   0.5810972
## 2  deviance -43.39425929 12.77751789 -66.452254705 -44.07871078 -16.3154741
## 3     mu[1]   0.03476251  0.11124472  -0.308933142   0.05814696   0.1593883
## 4     mu[2]   0.24000776  0.12834812   0.085632489   0.20764950   0.5716637
## 5 p.bias[1]   0.51442848  0.20049787   0.129091694   0.52680475   0.8609259
## 6 p.bias[2]   0.48557152  0.20049787   0.139074125   0.47319525   0.8709083
## 7       tau   0.15877664  0.05818935   0.062033759   0.15292163   0.2864157
##       Rhat
## 1 1.002403
## 2 1.001102
## 3 1.023437
## 4 1.002107
## 5 1.004029
## 6 1.001834
## 7 1.001711
##
## $K_12
##   Parameter          Mean          SD   CI_Lower_95       Median CI_Upper_95
## 1         B   0.206946828  0.15707957   0.008535266   0.17679304   0.5907419
```

```
## 2  deviance -43.217483420 12.78215468 -66.309844429 -43.85879490 -16.3908388
## 3     mu[1]  -0.004534213  0.14802832  -0.434208064   0.03876306   0.1496239
## 4     mu[2]   0.202412614  0.08465919   0.083988945   0.18585652   0.4131993
## 5 p.bias[1]   0.402679206  0.15782561   0.125292195   0.40140217   0.7079715
## 6 p.bias[2]   0.597320794  0.15782561   0.292028524   0.59859783   0.8747078
## 7       tau   0.131615010  0.04690939   0.052578377   0.12786311   0.2352080
##       Rhat
## 1 1.002984
## 2 1.001148
## 3 1.012451
## 4 1.001178
## 5 1.003444
## 6 1.002514
## 7 1.003756
##
## $K_9
##   Parameter          Mean          SD   CI_Lower_95       Median CI_Upper_95
## 1         B   0.188648772  0.15515326   0.007226059   0.15702414   0.5943314
## 2  deviance -43.353585733 12.73819488 -66.443032380 -43.95607774 -16.6085148
## 3     mu[1]  -0.001574383  0.14556454  -0.443912791   0.03592816   0.1485629
## 4     mu[2]   0.187074389  0.06732126   0.081064332   0.17755662   0.3457971
## 5 p.bias[1]   0.354924672  0.11181520   0.147593125   0.35267134   0.5784527
## 6 p.bias[2]   0.645075328  0.11181520   0.421547349   0.64732866   0.8524069
## 7       tau   0.122184770  0.03821325   0.054967684   0.11973377   0.2045738
##       Rhat
## 1 1.003781
## 2 1.000989
## 3 1.033383
## 4 1.000998
## 5 1.006466
## 6 1.003407
## 7 1.004758
##
## $K_5
##   Parameter          Mean          SD   CI_Lower_95       Median CI_Upper_95
## 1         B   0.182460203  0.13383052   0.007565469   0.15860496   0.4959454
## 2  deviance -43.050847493 12.79925707 -66.052181559 -43.76243996 -16.1726952
## 3     mu[1]  -0.007375801  0.11968643  -0.326970127   0.02123399   0.1435390
## 4     mu[2]   0.175084402  0.05333581   0.080534190   0.17121389   0.2908932
## 5 p.bias[1]   0.299079266  0.05631599   0.194449252   0.29729733   0.4147855
## 6 p.bias[2]   0.700920734  0.05631599   0.585214528   0.70270267   0.8055507
## 7       tau   0.111235272  0.03195145   0.053307764   0.10975530   0.1786622
##       Rhat
## 1 1.002184
## 2 1.001026
## 3 1.006766
## 4 1.001056
## 5 1.001042
## 6 1.001014
## 7 1.001467
```

## 2.6 Model Comparison (WAIC)

The Widely Applicable Information Criterion (WAIC) is computed to compare model fit. 1) Random Effects Model: WAIC is calculated using posterior samples of theta. 2) Bias Adjustment Models: WAIC is computed for each K value using theta.ba.

```r
# Function to compute log-likelihood for a normal distribution
compute_loglik <- function(y, mean, sd) {
  # y: observed data (vector of length N)
  # mean: posterior samples of the mean (S x N matrix, S = number of samples)
  # sd: standard deviation (vector of length N)
  # Returns: S x N matrix of log-likelihoods
```

```
  S <- nrow(mean)  # Number of posterior samples
  N <- length(y)   # Number of observations
  loglik <- matrix(NA, nrow = S, ncol = N)
  for (s in 1:S) {
    for (i in 1:N) {
      loglik[s, i] <- dnorm(y[i], mean = mean[s, i], sd = sd[i], log = TRUE)
    }
  }
  return(loglik)
}

# Function to compute WAIC from log-likelihood
compute_waic <- function(loglik) {
  # loglik: S x N matrix of log-likelihoods
  S <- nrow(loglik)  # Number of posterior samples
  N <- ncol(loglik)  # Number of observations

  # Compute lppd (log pointwise predictive density)
  lppd <- 0
  for (i in 1:N) {
    # Average the likelihood over posterior samples, then take log
    mean_lik <- mean(exp(loglik[, i]))
    lppd <- lppd + log(mean_lik)
  }

  # Compute p_WAIC (effective number of parameters)
  p_waic <- 0
  for (i in 1:N) {
    p_waic <- p_waic + var(loglik[, i])
  }

  # Compute WAIC
  waic <- -2 * (lppd - p_waic)
  return(list(waic = waic, lppd = lppd, p_waic = p_waic))
}

# Observed data
y_obs <- dat$g
se_y <- dat$se.y

# Extract posterior samples of theta[i] for Random Effects Model
theta_re <- jags_re$BUGSoutput$sims.list$theta  # S x N matrix

# Compute log-likelihood
loglik_re <- compute_loglik(y = y_obs, mean = theta_re, sd = se_y)

# Compute WAIC
waic_re <- compute_waic(loglik_re)

# Store results
waic_results <- data.frame(
  Model = "Random Effects",
  K_Label = NA_character_,
  WAIC = waic_re$waic,
  lppd = waic_re$lppd,
  p_waic = waic_re$p_waic
)

for (k_label in k_labels_vec) {

  # Extract JAGS object for the current K
  jags_ba_current <- jags_ba_list[[k_label]]
  if (is.null(jags_ba_current)) {
    warning(paste("JAGS object not found for", k_label, ". Skipping WAIC calculation."))
```

```
    next
  }

  # Extract posterior samples of theta.ba[i] for Bias Adjustment Model
  theta_ba <- jags_ba_current$BUGSoutput$sims.list$theta.ba  # S x N matrix
  if (is.null(theta_ba)) {
    warning(paste("theta.ba samples not found in JAGS object for", k_label,
                  ". Skipping WAIC calculation."))
    next
  }

  # Compute log-likelihood
  loglik_ba <- compute_loglik(y = y_obs, mean = theta_ba, sd = se_y)

  # Compute WAIC
  waic_ba <- compute_waic(loglik_ba)

  # Add to results
  waic_results <- rbind(waic_results, data.frame(
    Model = "Bias Adjustment",
    K_Label = k_label,
    WAIC = waic_ba$waic,
    lppd = waic_ba$lppd,
    p_waic = waic_ba$p_waic
  ))
}

# Update K_Label for better readability
waic_results <- waic_results %>%
  mutate(K_Label = case_when(
    K_Label == "K_16" ~ "K = 16",
    K_Label == "K_12" ~ "K = 12",
    K_Label == "K_9" ~ "K = 9",
    K_Label == "K_5" ~ "K = 5",
    TRUE ~ K_Label
  ))

# Print the WAIC results
print(waic_results)
```

```
##             Model K_Label     WAIC     lppd   p_waic
## 1  Random Effects    <NA> 2.380033 33.42408 34.61409
## 2 Bias Adjustment  K = 16 8.969076 33.65790 38.14244
## 3 Bias Adjustment  K = 12 9.203323 33.56292 38.16458
## 4 Bias Adjustment   K = 9 8.569089 33.54518 37.82973
## 5 Bias Adjustment   K = 5 9.340016 33.47425 38.14425
```

## 2.7 Posterior Distribution Plots

Posterior distributions for key parameters (mu[1], mu[2], tau, B, p.bias[2]) are visualized for the bias adjustment model across different K values. Due to storage constraints, plots are not displayed, and only the code is provided.

```
# Prepare posterior data
posterior_data <- bind_rows(posterior_ba, .id = "K_Label")

# Get unique K values and labels
k_labels <- unique(posterior_data$K_Label)
k_map <- setNames(beta_param$K, paste0("K_", beta_param$K))

# Assign specific K values to colors
k_values <- c("K = 16", "K = 12", "K = 9", "K = 5")
```

```
k_colors <- setNames(c("red", "blue", "green", "orange"), k_values)

# Update posterior_data to use these labels
posterior_data <- posterior_data %>%
  mutate(K_Label = case_when(
    K_Label == names(k_map)[1] ~ "K = 16",
    K_Label == names(k_map)[2] ~ "K = 12",
    K_Label == names(k_map)[3] ~ "K = 9",
    K_Label == names(k_map)[4] ~ "K = 5"
  ),
  K_Label = factor(K_Label, levels = k_values))

# Define parameter list and x-axis labels
params_to_plot <- c("mu1", "mu2", "tau", "B", "p.bias2")
param_labels_list <- list(
  mu1 = expression("Unbiased Mean (" * mu * ")"),
  mu2 = expression("Biased Mean (" * mu[biased] * ")"),
  tau = expression("Between-Study Heterogeneity (" * tau * ")"),
  B = expression("Bias Magnitude (" * B * ")"),
  p.bias2 = expression("Probability of Bias (" * pi[bias] * ")")
)

# Create individual plots and store them
plot_list_posterior <- list()

for (i in seq_along(params_to_plot)) {
  param <- params_to_plot[i]
  param_label <- param_labels_list[[param]]

  # Base plot
  p <- ggplot(posterior_data, aes(x = .data[[param]], color = K_Label)) +
    stat_density(geom = "line", position = "identity", linewidth = 0.7) +
    scale_color_manual(values = k_colors) +
    labs(
      x = param_label,
      y = "Posterior Density",
      color = "Median"
    ) +
    theme_minimal(base_size = 11) +
    theme(
      legend.position = "bottom",
      panel.grid = element_blank(),
      axis.line = element_line(color = "black"),
      plot.margin = margin(5, 20, 5, 5),
      axis.text = element_text(size = 11),
      axis.title = element_text(size = 11),
      legend.title = element_text(size = 11),
      legend.text = element_text(size = 11)
    )

  # Adjust scales based on parameter
  if (param %in% c("mu1", "mu2")) {
    p <- p +
      scale_x_continuous(limits = c(-1, 1), breaks = seq(-1, 1, by = 0.4), expand = c(0, 0)) +
      scale_y_continuous(limits = c(0, 10), breaks = seq(0, 10, by = 2), expand = c(0, 0))
  } else if (param == "tau") {
    p <- p +
      scale_x_continuous(limits = c(0, 0.5), breaks = seq(0, 0.5, by = 0.1), expand = c(0, 0)) +
      scale_y_continuous(limits = c(0, 15), breaks = seq(0, 15, by = 3), expand = c(0, 0))
  } else if (param == "B") {
    p <- p +
      scale_x_continuous(limits = c(-0.2, 1), breaks = seq(-0.2, 1, by = 0.2), expand = c(0, 0)) +
      scale_y_continuous(limits = c(0, 4), breaks = seq(0, 4, by = 1), expand = c(0, 0))
  } else if (param == "p.bias2") {
```

```
    p <- p +
      scale_x_continuous(limits = c(0, 1.0), breaks = seq(0, 1.0, by = 0.2), expand = c(0, 0)) +
      scale_y_continuous(limits = c(0, 8), breaks = seq(0, 8, by = 2), expand = c(0, 0))
  }

  plot_list_posterior[[param]] <- p
}

# Arrange mu[1] and mu[2] in a 1x2 grid
grid_mu <- grid.arrange(
  plot_list_posterior[["mu1"]],
  plot_list_posterior[["mu2"]],
  nrow = 1, ncol = 2,
  bottom = grid::textGrob("Median (K)", gp = grid::gpar(fontsize = 11))
)

# Print and save the mu[1] and mu[2] grid
print(grid_mu)

# Print and save individual plots for tau, B, and p.bias[2]
for (param in c("tau", "B", "p.bias2")) {
  p <- plot_list_posterior[[param]]
  print(p)
}
```

## 2.8 Posterior Predictive Checking

Posterior predictive checks compare observed data to replicated data under both models using density overlays and test statistics (mean and SD). Due to storage constraints, plots are not displayed, and only the code is provided.

```
# Set bayesplot theme
color_scheme_set("brightblue")
theme_set(theme_minimal(base_size = 11) +
            theme(panel.grid = element_blank(),
                  axis.line = element_line(color = "black"),
                  plot.margin = margin(10, 10, 10, 10),
                  plot.title = element_text(hjust = 0.5))
)

# Observed data
y_obs <- dat$g

# Extract y.rep samples
yrep_re <- jags_re$BUGSoutput$sims.list$y.rep

# 1. Density overlay plot
ppc_plot_re_dens <- ppc_dens_overlay(y_obs, yrep_re) +
  labs(title = "Posterior Predictive Density Overlay",
       x = "Effect Size (g)", y = "Density") +
  scale_x_continuous(limits = c(-3, 3), breaks = seq(-3, 3, by = 1), expand = c(0, 0))

# 2. Test statistics plots (Mean and SD)
ppc_plot_re_mean <- ppc_stat(y_obs, yrep_re, stat = "mean") +
  labs(title = "Test Statistic (Mean)") +
  scale_x_continuous(limits = c(0, 0.25), breaks = seq(0, 0.25, by = 0.05), expand = c(0, 0))

ppc_plot_re_sd <- ppc_stat(y_obs, yrep_re, stat = "sd") +
  labs(title = "Test Statistic (Standard Deviation)") +
  scale_x_continuous(limits = c(0.25, 0.6), breaks = seq(0.25, 0.6, by = 0.05), expand = c(0, 0))

# Combine RE plots without overall title
```

```
ppc_plots_re_combined <- ppc_plot_re_dens / (ppc_plot_re_mean + ppc_plot_re_sd)

# Print and Save RE plots
print(ppc_plots_re_combined)

ppc_ba_plots_list <- list()

for (k_label in k_labels_vec) {

  # Extract y.rep for the current K
  jags_ba_current <- jags_ba_list[[k_label]]
  if (is.null(jags_ba_current)) {
    warning(paste("JAGS object not found for", k_label, ". Skipping PPC."))
    next
  }
  yrep_ba <- jags_ba_current$BUGSoutput$sims.list$y.rep
  if (is.null(yrep_ba)) {
    warning(paste("y.rep samples not found in JAGS object for", k_label, ". Skipping PPC."))
    next
  }

  # 1. Density overlay plot
  ppc_plot_ba_dens <- ppc_dens_overlay(y_obs, yrep_ba) +
    labs(title = "Posterior Predictive Density Overlay",
         x = "Effect Size (g)", y = "Density") +
    scale_x_continuous(limits = c(-3, 3), breaks = seq(-3, 3, by = 1), expand = c(0, 0))

  # 2. Test statistics plots (Mean and SD)
  ppc_plot_ba_mean <- ppc_stat(y_obs, yrep_ba, stat = "mean") +
    labs(title = "Test Statistic (Mean)") +
    scale_x_continuous(limits = c(0, 0.25), breaks = seq(0, 0.25, by = 0.05), expand = c(0, 0))

  ppc_plot_ba_sd <- ppc_stat(y_obs, yrep_ba, stat = "sd") +
    labs(title = "Test Statistic (Standard Deviation)") +
    scale_x_continuous(limits = c(0.25, 0.6), breaks = seq(0.25, 0.6, by = 0.05), expand = c(0, 0))

  # Combine plots for this K without overall title
  ppc_plots_ba_combined <- ppc_plot_ba_dens / (ppc_plot_ba_mean + ppc_plot_ba_sd)

  # Store the combined plot
  ppc_ba_plots_list[[k_label]] <- ppc_plots_ba_combined

  # Print and Save BA plots for this K
  print(ppc_plots_ba_combined)
}
```

### 2.9 Reporting Results and Visualization

Combine results and create forest plots

```
## Create the combined results
# Combine RE summary with a model identifier
results_re_df_combined <- results_re_df %>%
  mutate(Model = "RE", K_Label = NA_character_)

# Combine all BA summaries from the results_ba list
results_ba_combined_list <- lapply(names(results_ba), function(k_label) {
  df <- results_ba[[k_label]]
  df <- df %>%
    mutate(Model = "BA", K_Label = k_label)
  return(df)
})
```

```
# Bind all results into a single data frame
results_all_combined <- bind_rows(
  results_re_df_combined,
  bind_rows(results_ba_combined_list)
)

## Create study-specific forest plot
# For random effect model (theta)
re_theta_summary <- jags_re$BUGSoutput$summary[grepl("^theta\\[", rownames(jags_re$BUGSoutput$summary))
re_theta_df <- data.frame(
  Study = dat$study,  # Assumes 'dat' has a 'study' column
  Model = "RE",
  K_Label = NA_character_,
  Mean = re_theta_summary[, "mean"],
  CI_Lower_95 = re_theta_summary[, "2.5%"],
  CI_Upper_95 = re_theta_summary[, "97.5%"]
)

# For bias adjustment models (theta.ba)
ba_theta_list <- lapply(names(jags_ba_list), function(k_label) {
  ba_theta_summary <- jags_ba_list[[k_label]]$BUGSoutput$summary[grepl("^theta\\.ba\\[", rownames(jags_
  df <- data.frame(
    Study = dat$study,
    Model = "BA",
    K_Label = k_label,
    Mean = ba_theta_summary[, "mean"],
    CI_Lower_95 = ba_theta_summary[, "2.5%"],
    CI_Upper_95 = ba_theta_summary[, "97.5%"]
  )
  return(df)
})

# Combine all theta estimates
theta_all <- bind_rows(
  re_theta_df,
  bind_rows(ba_theta_list)
)

# Add a combined Model_K column for plotting with custom labels
theta_all <- theta_all %>%
  mutate(Model_K = case_when(
    Model == "RE" ~ "Random Effect",
    Model == "BA" & K_Label == "K_16" ~ "Bias Adjustment (K=16)",
    Model == "BA" & K_Label == "K_12" ~ "Bias Adjustment (K=12)",
    Model == "BA" & K_Label == "K_9" ~ "Bias Adjustment (K=9)",
    Model == "BA" & K_Label == "K_5" ~ "Bias Adjustment (K=5)"
  )) %>%
  mutate(Model_K = factor(Model_K, levels = c("Random Effect",
                                              "Bias Adjustment (K=16)",
                                              "Bias Adjustment (K=12)",
                                              "Bias Adjustment (K=9)",
                                              "Bias Adjustment (K=5)")))

# Create the forest plot with smaller dot size
specific_forest_plot <- ggplot(theta_all, aes(x = Study, y = Mean,
                                              ymin = CI_Lower_95, ymax = CI_Upper_95, color = Model_K))
  geom_pointrange(position = position_dodge(width = 0.5), size = 0.3) +
  coord_flip() +
  labs(
    x = "Study",
    y = "Effect Size (95% Credible Interval)",
    color = "Model"
  ) +
  scale_y_continuous(limits = c(-1.5, 2.5), breaks = seq(-1.5, 2.5, by = 0.5)) +
```

```
  theme_minimal(base_size = 11) +
  theme(
    legend.position = "bottom",
    axis.text.y = element_text(size = 8),
    panel.grid.major = element_blank(),
    panel.grid.minor = element_blank(),
    axis.line = element_line(color = "black")
  ) +
  scale_color_manual(values = c("Random Effect" = "black",
                                "Bias Adjustment (K=16)" = "red",
                                "Bias Adjustment (K=12)" = "blue",
                                "Bias Adjustment (K=9)" = "green",
                                "Bias Adjustment (K=5)" = "orange"))

# Print the plot
print(specific_forest_plot)
```

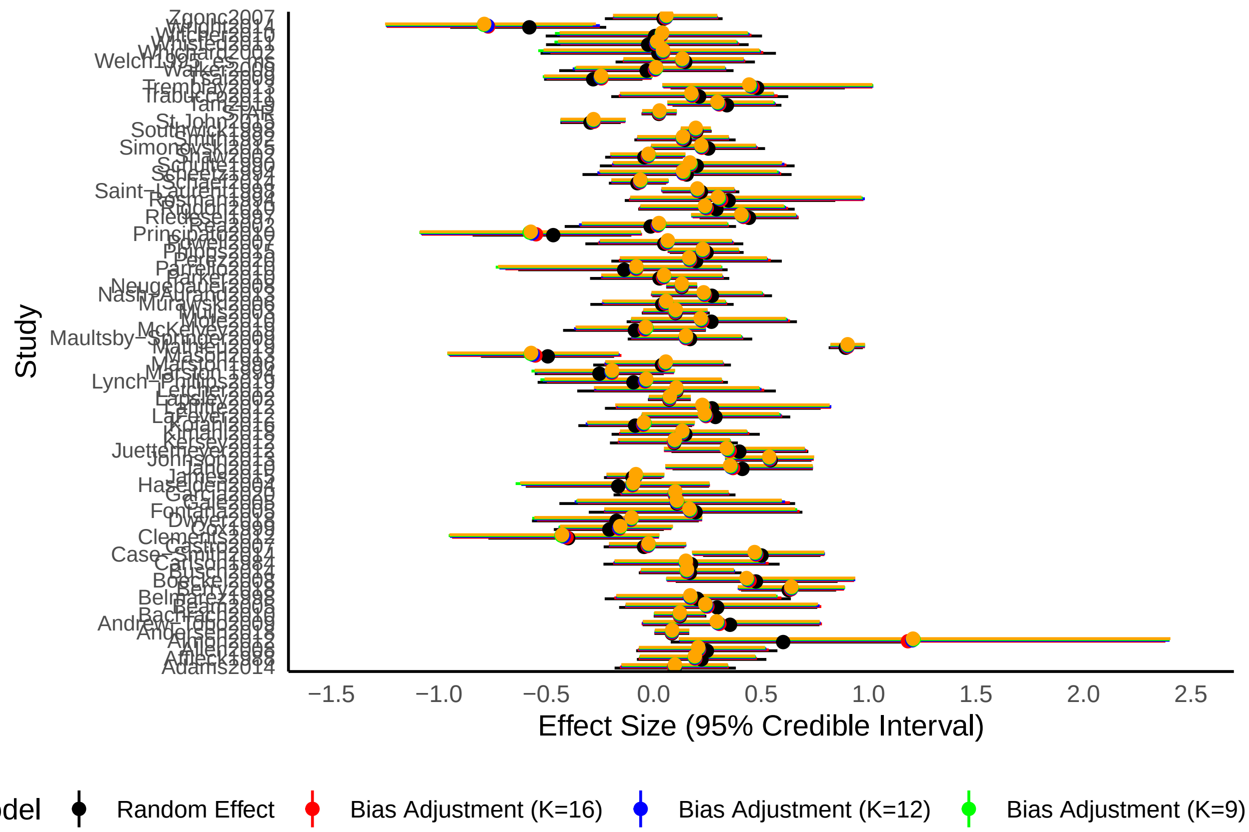


```
## Create overall study forest plot by model
# From random effect model (mu)
re_overall <- results_re_df %>%
  filter(Parameter == "mu") %>%
  select(Mean, CI_Lower_95, CI_Upper_95) %>%
  mutate(Model = "Random Effect", K_Label = NA_character_, Parameter = "Overall Effect")

# From bias adjustment models (mu[1] and mu[2])
ba_overall_list <- lapply(names(results_ba), function(k_label) {
  df <- results_ba[[k_label]] %>%
    filter(Parameter %in% c("mu[1]", "mu[2]")) %>%
    select(Parameter, Mean, SD, CI_Lower_95, Median, CI_Upper_95, Rhat) %>%
    mutate(Model = paste("Bias Adjustment (K=", sub("K_", "", k_label), ")", sep = ""),
```

```
           K_Label = k_label)
  return(df)
})

# Combine all overall effect sizes
overall_all <- bind_rows(
  re_overall,
  bind_rows(ba_overall_list)
)

# Add a type column to distinguish Unbiased (mu[1]) and Biased (mu[2])
overall_all <- overall_all %>%
  mutate(Type = case_when(
    Parameter == "mu[1]" ~ "Unbiased",
    Parameter == "mu[2]" ~ "Biased",
    Model == "Random Effect" ~ "Overall"
  )) %>%
  mutate(Model = factor(Model, levels = rev(c("Random Effect",
                                              "Bias Adjustment (K=16)",
                                              "Bias Adjustment (K=12)",
                                              "Bias Adjustment (K=9)",
                                              "Bias Adjustment (K=5)")))) %>%
  mutate(Type = factor(Type, levels = c("Overall", "Unbiased", "Biased")))

overall_forest_plot <- ggplot(overall_all, aes(y = Model, color = Type)) +
  geom_errorbar(aes(xmin = CI_Lower_95, xmax = CI_Upper_95), width = 0, size = 1,
              position = "identity") +
  geom_segment(aes(x = CI_Lower_95, xend = CI_Lower_95, y = as.numeric(Model) - 0.1,
                   yend = as.numeric(Model) + 0.1), size = 1) +
  geom_segment(aes(x = CI_Upper_95, xend = CI_Upper_95, y = as.numeric(Model) - 0.1,
                   yend = as.numeric(Model) + 0.1), size = 1) +
  geom_point(aes(x = Mean), size = 3.5, shape = 16) +
  geom_vline(xintercept = 0, linetype = "dashed", color = "gray50") +
  labs(
    x = "Overall Effect Size (95% Credible Interval)",
    y = "Model",
    color = "Effect Type"
  ) +
  scale_x_continuous(limits = c(-0.5, 0.8), breaks = seq(-0.5, 0.8, by = 0.1)) +
  theme_minimal(base_size = 11) +
  theme(
    legend.position = "bottom",
    legend.text = element_text(size = 11),
    legend.title = element_text(size = 11),
    axis.text.x = element_text(size = 11),
    axis.text.y = element_text(size = 11),
    axis.title.x = element_text(size = 11),
    axis.title.y = element_text(size = 11),
    panel.grid.major = element_blank(),
    panel.grid.minor = element_blank(),
    axis.line = element_line(color = "black"),
    plot.margin = margin(5, 5, 5, 5)
  ) +
  scale_color_manual(values = c("Overall" = "orange",
                                "Unbiased" = "blue",
                                "Biased" = "red"))
# Print the plot
print(overall_forest_plot)
```

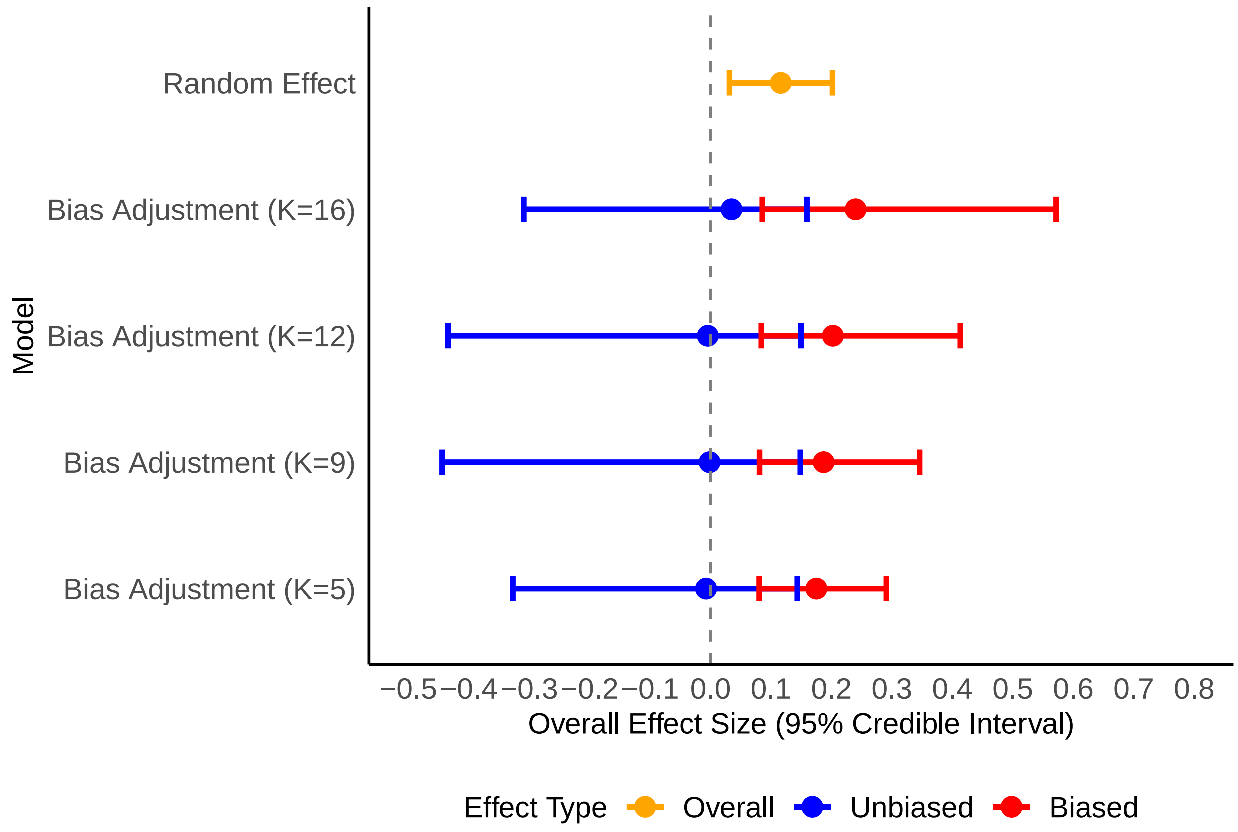


## 3 Simulation Data Analysis

The `sim_rob_data` function generates simulated meta-analysis data with specified risk of bias proportions, effect sizes, and heterogeneity.

```
# "sim_rob_data" function to generate simulated data for a meta-analysis with studies classified by ris
# seed: seed for reproducibility
# k: number of studies
# prop_low: proportion of low risk-of-bias studies
# prop_unclear: proportion of unclear risk-of-bias studies
# prop_high: proportion of high risk-of-bias studies
# theta: true unbiased effect size
# tau: between-study heterogeneity standard deviation
# bias_mean: mean bias for biased studies
# df_chisq: degrees of freedom for chi-squared sample size generation
# scale_n: scaling factor for sample sizes
# min_n: minimum sample size

sim_rob_data <- function(seed,
                         k, prop_low, prop_unclear, prop_high,
                         theta, tau, bias_mean,
                         df_chisq, scale_n, min_n) {

  # Step 1: Validate proportions
  prop_sum <- prop_low + prop_unclear + prop_high
  if (abs(prop_sum - 1) > 1e-6) {
    stop("Proportions (prop_low, prop_unclear, prop_high) must sum to 1. Got sum = ", prop_sum)
  }
```

```
# Set seed for reproducibility
set.seed(seed)

# Step 2: Calculate number of studies per risk-of-bias category
n_low <- round(k * prop_low)
n_unclear <- round(k * prop_unclear)
n_high <- k - n_low - n_unclear

# Step 3: Generate sample sizes
n <- ceiling(rchisq(k, df = df_chisq) * scale_n + min_n)
n1 <- ceiling(n / 2)
n2 <- floor(n / 2)
n_tilde <- (n1 * n2) / (n1 + n2)

# Step 4: Generate initial Hedges' g (unbiased)
x <- rnorm(k, theta, sqrt(2 / n))
S <- rchisq(k, 2 * n - 2, ncp = 0) / (2 * n - 2)
g_initial <- x / sqrt(S)
g_initial <- pmin(pmax(g_initial, -3), 3)

# Step 5: Assign risk-of-bias levels
risk_levels <- c(rep("low", n_low), rep("unclear", n_unclear), rep("high", n_high))
risk_levels <- sample(risk_levels)

# Step 6: Generate bias indicators (I[i])
I <- numeric(k)
for (i in 1:k) {
  if (risk_levels[i] == "low") {
    I[i] <- 0
  } else if (risk_levels[i] == "high") {
    I[i] <- 1
  } else {
    I[i] <- rbinom(1, 1, 0.5)
  }
}

# Step 7: Generate biased Hedges' g
g_biased <- numeric(k)
for (i in 1:k) {
  if (I[i] == 0) {
    g_biased[i] <- g_initial[i]
  } else {
    q_i <- rbeta(1, 0.5, 1)
    tau_beta_i <- tau * sqrt(1 / q_i - 1)
    beta_i <- rnorm(1, bias_mean, tau_beta_i)
    g_biased[i] <- g_initial[i] + beta_i
  }
}

# Step 8: Add between-study heterogeneity
u <- rnorm(k, 0, tau)
d <- g_biased + u

# Step 9: Calculate variance (standard Hedges' g)
vm <- 1 / n_tilde + g_biased^2 / (2 * (n1 + n2 - 2))
se_y <- sqrt(vm)
se_y <- pmin(se_y, quantile(se_y, 0.95))

# Step 10: Compile simulation data
sim_data <- data.frame(
  study_id = 1:k,
  y = d,
  se_y = se_y,
  risk_level = risk_levels,
```

```
    I = I,
    g_true = g_initial,
    g_B_true = g_biased
  )

  return(sim_data)
}

# Generate simulation data using "sim_rob_data" function
sim_data <- sim_rob_data(seed = 2025,
                         k = 100, prop_low = 0.03, prop_unclear = 0.43, prop_high = 0.54,
                         theta = 0.0, tau = 0.3, bias_mean = 0.2,
                         df_chisq = 5, scale_n = 20, min_n = 20)
```
